# MicroRNA–mRNA interactions underlying colorectal cancer molecular subtypes

**DOI:** 10.1038/ncomms9878

**Published:** 2015-11-17

**Authors:** Laura Cantini, Claudio Isella, Consalvo Petti, Gabriele Picco, Simone Chiola, Elisa Ficarra, Michele Caselle, Enzo Medico

**Affiliations:** 1 Department of Oncology, Università degli Studi di Torino, S.P. 142, km 3, 95—10060 Candiolo, Italy; 2 Department of Control and Computer Engineering, Politecnico di Torino, Cso Duca degli Abruzzi 24, 10129 Torino, Italy; 3 Istituto Nazionale Biostrutture e Biosistemi—Consorzio Interuniversitario, Viale delle Medaglie d'Oro, 305—00136 Roma, Italy; 4 Candiolo Cancer Institute, FPO IRCCS, S.P. 142, km 3, 95—10060 Candiolo, Italy; 5 Department of Physics and INFN, Università degli Studi di Torino, via P.Giuria 1, I-10125 Turin, Italy

## Abstract

Colorectal cancer (CRC) transcriptional subtypes have been recently identified by gene expression profiling. Here we describe an analytical pipeline, microRNA master regulator analysis (MMRA), developed to search for microRNAs potentially driving CRC subtypes. Starting from a microRNA–mRNA tumour expression data set, MMRA identifies candidate regulator microRNAs by assessing their subtype-specific expression, target enrichment in subtype mRNA signatures and network analysis-based contribution to subtype gene expression. When applied to a CRC data set of 450 samples, assigned to subtypes by 3 different transcriptional classifiers, MMRA identifies 24 candidate microRNAs, in most cases downregulated in the stem/serrated/mesenchymal (SSM) poor prognosis subtype. Functional validation in CRC cell lines confirms downregulation of the SSM subtype by miR-194, miR-200b, miR-203 and miR-429, which share target genes and pathways mediating this effect. These results show that, by combining statistical tests, target prediction and network analysis, MMRA effectively identifies microRNAs functionally associated to cancer subtypes.

## Abstract (web summary)

Colorectal cancer subtypes can be distinguished by their different biological and molecular properties. Here the authors present microRNA Master Regulator Analysis, a tool to identify microRNAs driving subtype-specific gene expression and cancer variation.

## Graphical abstract

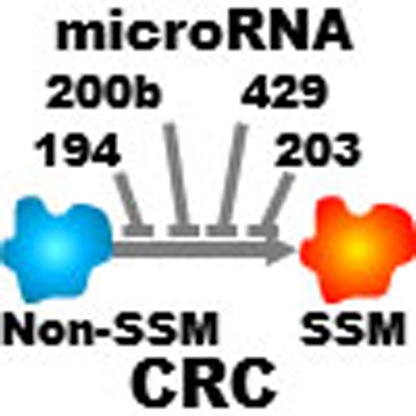

Colorectal Cancer (CRC) is a major cause of cancer mortality and is endowed with wide molecular, biological and clinical heterogeneity. Recently, multiple research groups have independently identified transcriptional signatures defining CRC molecular subtypes, endowed with different biological properties (crypt cell subtype, active pathways), molecular features (type of genomic instability, oncogenic mutations and methylator phenotype) and clinical features (prognosis, response to treatment)^1^,^2^,^3^,^4^,^5^. The number of distinct subtypes identified ranges from three to six, which raised the question of what are the correlations between the subtypes defined in the different works. Recently, we provided a unifying frame to reconcile the different CRC classification systems^6^. The consensus partition is composed of three major transcriptional categories: (1) inflammatory/goblet; (2) TA/enterocyte and (3) stem/serrated/mesenchymal (SSM). A still pending issue is which biological mechanisms and regulatory networks underlie the CRC molecular subtypes. In this context, a key role may be played by microRNAs, small non-coding RNAs of 20–22 nucleotides that bind complementary sequences in target mRNAs and thus reduce their stability and translation rate^7^. Indeed, several microRNAs have been shown to have altered expression associated to pro-oncogenic or tumour suppressor activity in CRC^8^. In particular, a number of so-called oncomiRs have been identified for their ability to influence key steps in the metastatic process and to be involved in circuits-regulating epithelial to mesenchymal transition (EMT), a critical step that drives tumour metastasis. It is therefore reasonable to hypothesize that some microRNAs may have a driving role on the CRC transcriptional subtypes. Identification of such microRNAs requires integrative analysis of paired microRNA–mRNA expression profiles from a large set of CRC samples. Recently, integrative computational methods have been proposed to discover microRNA–mRNA interactions possibly involved in tumour development^8^,^9^. However, these methods have been typically applied to distinguish tumour from normal tissue, a comparison characterized by much wider variation than between two tumour subtypes. Moreover, the methods only take into account microRNA–mRNA interactions supported by anticorrelation, while it has been recently observed that microRNAs can act also indirectly through, for example, regulation of silencing complexes^10^. Finally, the above methods do not prioritize the identified microRNA–mRNA interactions.

To overcome all these limitations, we propose the microRNA master regulator analysis (MMRA) analysis pipeline, aimed at discovering which microRNAs potentially regulate which CRC subtype. MMRA is subdivided in four sequential steps, each aimed at progressively reducing the number of candidate microRNAs: (i) differential expression analysis to highlight microRNAs with subtype-specific expression; (ii) target transcript enrichment analysis, to further select those microRNAs whose predicted targets are enriched in the associated subtype mRNA signature; (iii) network analysis, in which an mRNA network is constructed around each microRNA using ARACNE (ref. 11), and tested for enrichment in signature genes; (iv) identification of microRNAs whose expression ‘explains' the expression of subtype signature genes, using stepwise linear regression (SLR) analysis^12^. An overview of the workflow and of the algorithmic steps is provided in Fig. 1. Here MMRA is first applied to CRC samples subdivided by their microsatellite instability status, where it promptly identifies microRNAs known to be associated with this molecular phenotype. MMRA is then applied to a paired mRNA–microRNA expression data set of 450 CRC samples whose transcriptional subtype, according to three different classifiers, is already established^6^. In this data set MMRA identifies several microRNAs whose increased expression is associated with downregulation of SSM subtype genes. MicroRNA–mRNA associations involved in subtype determination are confirmed in a CRC cell line mRNA–microRNA expression data set, and functionally validated by microRNA silencing experiments *in vitro*. These results show the efficacy of MMRA in identifying microRNAs functionally associated to cancer subtypes, with diagnostic and therapeutic implications.

## Results

### Expression data set assembly and preliminary tests

For this study, we obtained CRC microRNA expression data from The Cancer Genome Atlas (TCGA), and generated a matched mRNA–microRNA expression data set by integrating mRNA expression data that we previously assembled from TCGA^6^ for the same samples. Transcriptional classification of the samples according to the three above mentioned classifiers, dubbed colon cancer subtypes (CCS^1^), CRC-assigner (CRCA^2^) and colon cancer molecular subtypes (CCMS^3^) was obtained from Supplementary Table 1 of Isella *et al.*^6^ Notably, all three signatures classify the large majority of the TCGA samples with high statistical confidence (false discovery rate (FDR) <5%): 94% for CCMS, 90% for CRCA and 74% for CCS. However, definition of the optimal transcriptional classification of CRC into molecular subtypes is still an ongoing process, which brings some degree of uncertainty about the underlying mRNA–microRNA networks. To test MMRA on an unambiguous phenotype, we applied it to CRC samples subdivided by their microsatellite instability status, which was available for 280 samples of the data set. As MMRA uses in steps (ii–iv) a possibly independent mRNA signature distinguishing sample subgroups, we adopted a published signature composed of 53 mRNAs upregulated in microsatellite stable (MSS) samples and 11 mRNAs upregulated in microsatellite instable (MSI) samples^13^. MMRA identified three microRNAs potentially regulating the MSI/MSS transcriptome, two upregulated in MSS (miR-196b and miR-106a) and one upregulated in MSI samples (miR-31). Indeed, the role of all three microRNAs in regulating the MSI/MSS phenotype is well documented and in accordance with our findings^14^,^15^,^16^, which confirms the validity of the approach.

### Application of MMRA to CRC transcriptional subtypes

**Figure 2:**
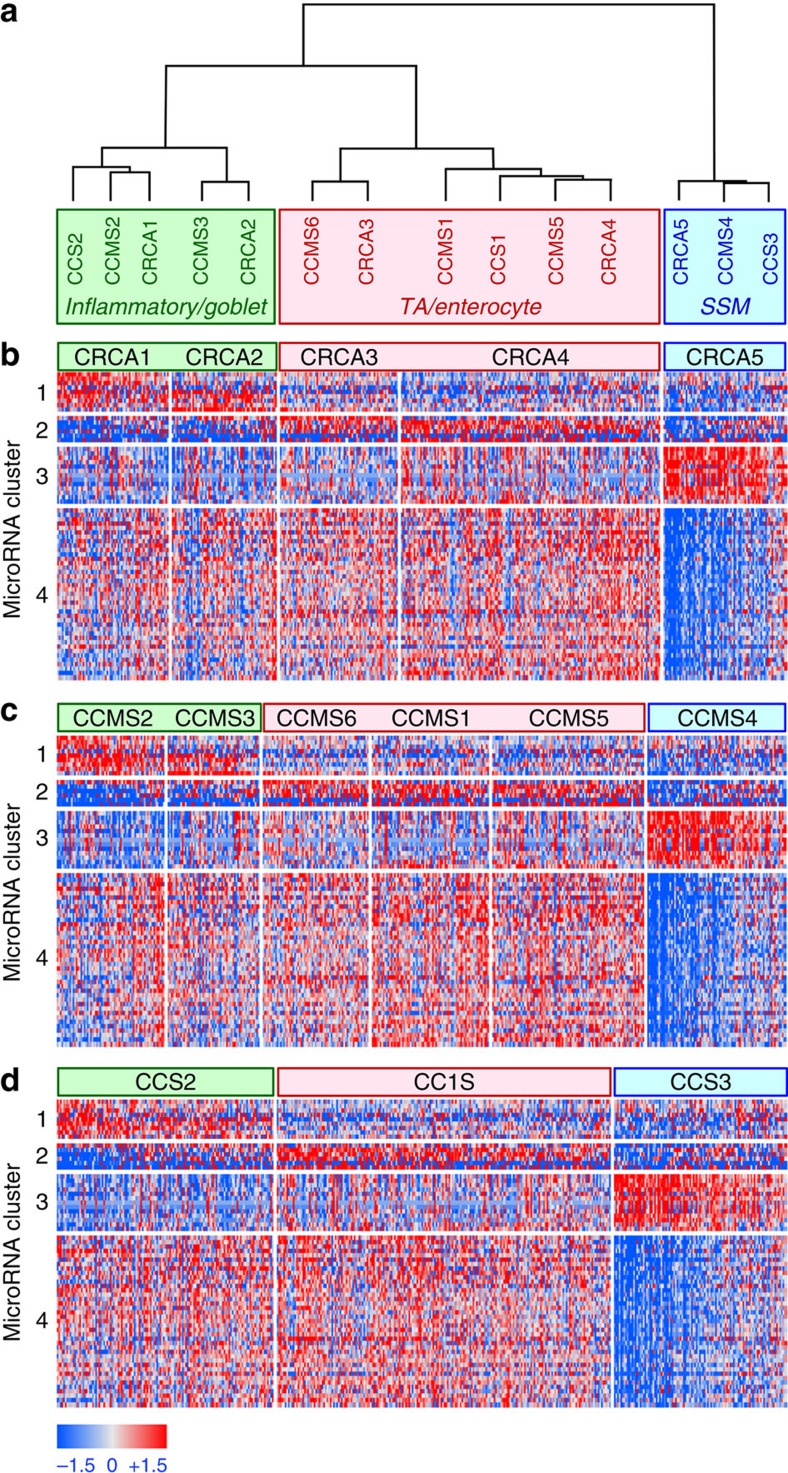
Subtypes consensus clustering applied to differentially expressed microRNAs in TCGA data set. (**a**) Consensus hierarchical clustering of 14 subtype centroids (CRCA 1–5, CCS 1–3 and CCMS 1–6). Each centroid was calculated by averaging, for each of 66 microRNAs differentially expressed in at least one subtype, expression in the samples assigned to the subtype. The dendrogram shows a subdivision of the subtype centroids in three major subgroups: SSM (blue), TA/enterocyte (red) and inflammatory/goblet (green). (**b**–**d**) Heatmaps displaying the expression of the 66 subtype-specific microRNAs in samples subdivided by, respectively, the CRCA (**b**), CCMS (**c**) and CCS (**d**) classifiers. MicroRNAs are subdivided by fuzzy self-organizing maps in four expression clusters with differential expression across the three consensus subgroups.

The first step of the pipeline consisted of finding microRNAs with subtype-specific expression. The results are summarized in Supplementary Data Set 1. In particular, we detected 52 microRNAs differentially expressed across CCS subtypes, with a FDR of 0.1%, 59 across CRCA subtypes (FDR=0.2%) and 54 across CCMS subtypes (FDR=0.7%). The analysis revealed a considerable overlap in differential microRNAs (Supplementary Fig. 1): 44 microRNAs displayed subtype-specific expression in all 3 classifiers, and only 11 were significant for just 1 classifier. Such a wide overlap suggested that specific subtypes from the various classifiers do indeed share the same upregulated or downregulated microRNAs. To provide a unified view of subtype-specific microRNA expression across all three classifiers, and possibly build a microRNA-based subtype consensus, we selected all the microRNAs differentially expressed in at least one subtype of at least one classifier (in total, 66 microRNAs). We then computed 14 centroids, considering the mean expression of these microRNAs in each subtype of each classifier (CRCA 1–5, CCS 1–3 and CCMS 1–6). To this centroid matrix we applied a consensus hierarchical clustering with *P* values (pvclust R package^17^). The resulting hierarchical tree (Fig. 2a and Supplementary Fig. 2) highlighted a first subdivision between SSM and non-SSM centroids with a confidence >95%. The non-SSM centroids were then further partitioned in two subgroups: TA/enterocyte and inflammatory/goblet. These results are in complete accordance with our previous subtype reconciliation based on mRNA expression^6^, highlighting a strong correlation between mRNA- and microRNA-based transcriptional classification of CRC. Figure 2 shows expression of the 66 microRNAs in CRC subtypes assigned by the 3 classifiers, organized by the hierarchical subtype consensus. MicroRNA clustering by their expression across all samples highlighted four major classes, respectively (i) upregulated in inflammatory/goblet; (ii) upregulated in TA/enterocyte; (iii) upregulated in SSM; and (iv) downregulated in SSM. The size of the clusters clearly shows that most of the microRNAs are differentially expressed between SSM and non-SSM subtypes.

**Figure 3:**
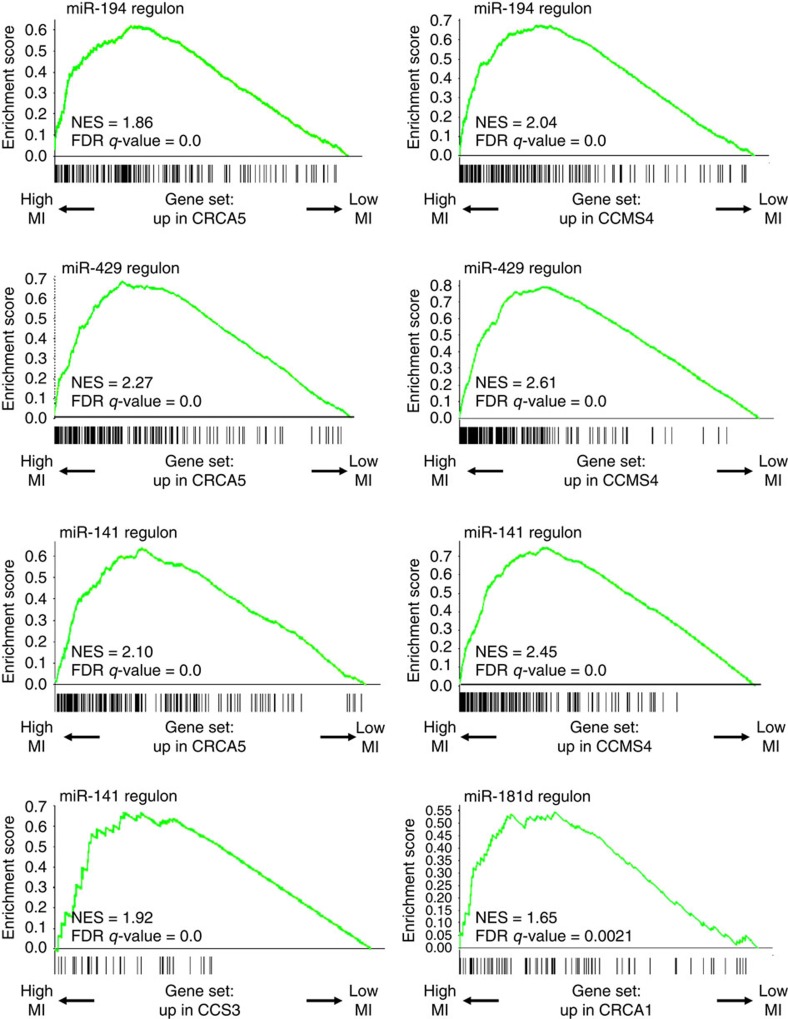
CRC subtype signature genes have high MI with specific microRNAs. The figure reports GSEA analysis of CRC subtype signatures within selected microRNA regulons, as indicated on top of each panel. The signatures were selected among those enriched in genes contained in the regulon. Within each of the indicated microRNA regulons, genes are sorted by decreasing MI with the microRNA, from left to right. The enrichment plots show that the displayed signatures are also enriched in genes with particularly high MI with the microRNA within the regulon.

In the second step of the pipeline, 31 of the 66 subtype-specific microRNAs were found to also have their predicted targets enriched in genes of the corresponding subtype mRNA signature (some of them in more than 1 signature and/or more than 1 classifier). The results of this step are reported in Supplementary Table 1. In the third MMRA step, a ‘regulon' (a single-hub network of significant interactions) was first constructed around each of the 31 selected microRNAs using the paired mRNA expression data set (see Methods section). The number of links in the obtained regulons varied widely, from 17 to 1,492 mRNAs, however being between 300 and 600 in the majority of the cases. In each regulon, mutual information (MI) values were almost invariably between 0.11 and 0.4. Details are reported in Supplementary Table 2. Subsequently, the 31 regulons were tested for enrichment in subtype signature genes. As a result, only one microRNA was filtered out, confirming a good correspondence between the subtype-based analysis of steps i and ii, and the unsupervised network-based approach of step iii. Remarkably, in some cases, signature genes were not only enriched in the regulons, but also, within the regulon, were among those with the highest MI values. Figure 3 reports four such cases (miR-194, miR-429, miR-141 and miR-181d). A summary of all the results of this step is reported in Supplementary Table 3. The fourth step of MMRA further restricted the candidate microRNAs to 24 whose expression was found to fit the expression of subtype signature genes included in their regulons, according to SLR analysis^12^,^18^. For the majority of them (20) expression of the microRNA was opposite to that of the associated gene signature, while the remaining 4 had concordant expression.

The final output of the MMRA pipeline is reported in Table 1. Interestingly, 16 out of the 24 identified microRNAs were negatively associated to the SSM subtype: they had lower expression in CCMS4, CRCA5 and CCS3 samples and were associated by the pipeline to genes upregulated in the same subtypes. Therefore, most of the MMRA-identified microRNAs are likely regulating a more generic ‘SSM/non-SSM' subdivision, rather than driving single subtypes. This result is in line with the major bifurcation observed between SSM and non-SSM samples described in Fig. 2.

### Validation of microRNA-subtype associations in cell lines

We recently found that genes whose expression is positively associated with the SSM subgroup of CRC are mostly expressed by stromal cells^6^. Nevertheless, in an expression data set of 151 CRC cell lines, we detected the SSM subtype with confidence in about 15% of the cases^19^. This indicates that in some cases CRC neoplastic cells do indeed undergo epithelial–mesenchymal transition and display stem cell-like features, and that exploiting mRNA–microRNA interactions may help distinguishing cancer cell-intrinsic features from stromal contribution. Therefore, to test whether microRNA–mRNA interactions highlighted by the pipeline hold true also in the absence of stromal cells, we assembled a paired mRNA–microRNA expression data set consisting of 18 CRC cell lines for which we had generated both mRNA and microRNA expression profiles. To test microRNA differential expression across subtypes, we took advantage of our previous subtype assignment for the 18 cell lines^19^. Considering subtype-specific expression, almost all microRNAs identified by the MMRA pipeline agreed in cell lines with the upregulation or downregulation observed in the TCGA data set (11/13 in CCMS, 16/19 in CRCA and 2/2 in CCS), confirming the reliability of the cell lines as model for the transcriptional subtypes. We then performed a more stringent analysis of microRNA-subtype association in CRC cell lines, for which validated microRNAs had to fulfil two conditions: (i) TCGA-concordant differential expression of the microRNA between cell lines of the target subtype and all other cell lines, beyond a significance threshold of 1.321722 for CCMS, 1.237681 for CRCA and 1.521026 for CCS; (ii) significant fraction of subtype signature genes (5% for CCMS and 10% for CRCA) whose expression is correlated (positively or negatively) to the microRNA across subtypes. More details are provided in Methods section. MicroRNA/subtype association were maintained in cell lines according to both criteria for 7 (30%) of the 24 microRNAs. The fraction of validated associations increased to 38% (5 of 13) for those considering only the CCMS classifier, for which subtype gene signatures have the largest size. Considering validation in cell lines, MMRA was found to be substantially more reliable than alternative pipelines developed to detect microRNA–mRNA interactions dysregulated in cancer versus normal tissue^8^,^9^. Similarly, simpler, less computationally intensive versions of the MMRA pipeline gave less reliable results. More details about these comparisons, including tests of validation in independent data sets, are provided in Supplementary Note.

### MicroRNA functional validation in cell lines

To functionally validate the identified associations between microRNAs and CRC subtypes, we prioritized three cell lines in which, based on mRNA and microRNA expression data, downregulation of specific microRNAs that are upregulated in non-SSM samples should result in a transcriptional shift towards the SSM subtype. In particular, NCIH508 cells were selected for validation of four microRNAs (miR-194, miR-200b, miR-203 and miR-429), HT29 cells for three microRNAs (miR-194, miR-200b and miR-429) and SW403 cells for miR-429. Details about the procedures used for microRNA and cell line prioritization are provided in Methods section. Cells were transduced with lentiviral vectors carrying microRNA-targeting sequences (‘miRZIPs') to downregulate expression of each individual microRNA. After selection of stably transduced cells, microarray-based mRNA expression profiling was conducted to evaluate transcriptional changes. In accordance with the above-described subtype consensus and MMRA output, we considered only two major cellular states: SSM and non-SSM. To obtain two complementary gene signatures for this partition, we grouped together on one side all the SSM-UP subtype signatures, and on the other all the SSM-DOWN subtype signatures (details are reported in Methods section). Two tests were made to prove the driver role of the four microRNAs: (i) enrichment analysis of SSM-UP/DOWN signature genes within the sets of genes that were upregulated or downregulated on microRNA silencing; and (ii) assignment of the transduced versus control cell lines to the SSM- or non-SSM classes using nearest template prediction (NTP)^20^, a class prediction algorithm with confidence assessment that we previously used to classify CRC samples and cell lines^6^,^19^. The results of the tests are summarized in Table 2. Details about gene modulation after mirZIPs transduction are provided in Supplementary Data Sets 2–4, respectively, for HT29, NCIH508 and SW403 cell lines. Remarkably, as hypothesized, silencing of the selected microRNAs induced a detectable transcriptional shift towards the stem-like state in all cases. In particular, genes upregulated by mirZIPs were significantly enriched in SSM-UP genes in seven out of eight cases, and genes downregulated by mirZIPs were significantly enriched in SSM-DOWN genes in five out of eight cases, with at least one of the two enrichment tests significant in all cases. Moreover, in all cases but one, mirZIP transduction led to NTP-based reassignment of the cells to the SSM subtype with confidence. A possible explanation for the only exception is that SW403 cells are strongly non-SSM. Therefore, despite a strong significance of both SSM-UP and SSM-DOWN enrichment analyses, they failed to reprogram to the SSM phenotype.

**Figure 4:**
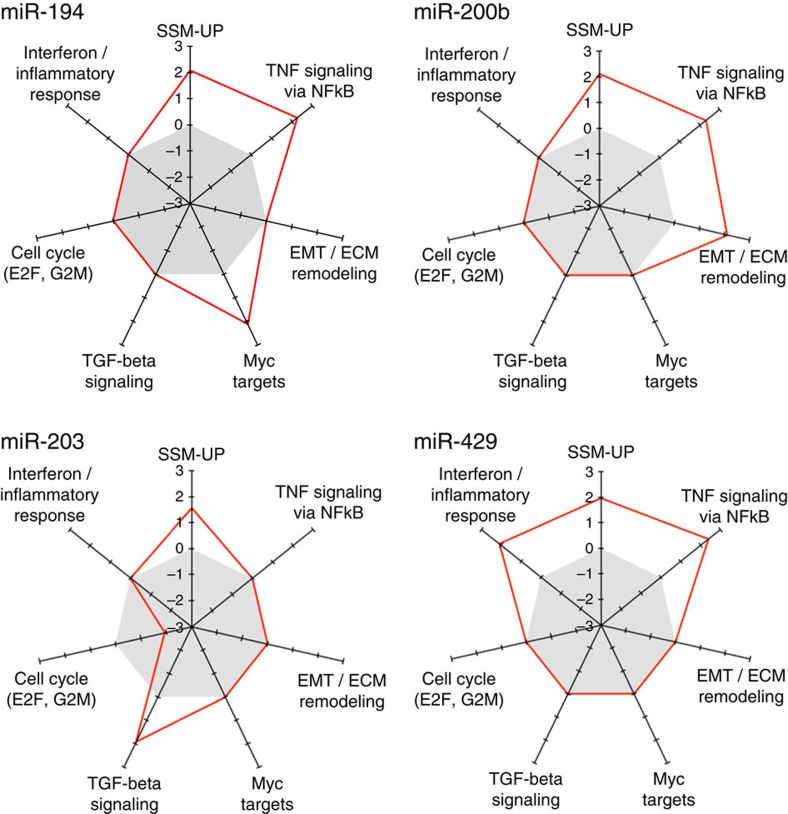
Transcriptional responses to microRNA downregulation in CRC cell lines. Radar plots representing transcriptional modulation of functional gene sets during the response of CRC cell lines to downregulation of, respectively, miR-194, miR-200b, miR-203 and miR-429 as indicated. The axes report the GSEA normalized enrichment scores (NES) for functional gene sets significantly enriched in at least one microRNA downregulation experiment. The grey area indicates a negative NES, meaning that the gene set is downregulated by microRNA silencing, while positive NES indicates gene set upregulation by microRNA silencing.

To identify the signalling pathways modulated in cell lines on silencing of the various microRNAs by their respective mirZIPs, we applied gene set enrichment analysis (GSEA)^21^ to the whole set of expressed genes ranked by their differential expression in mirZIP-transduced versus control cells. When a mirZIP was used in more than one cell line, differential expression values were averaged. The same GSEA analysis was also applied to the above-described SSM-UP signature, to verify enrichment of upregulated genes in a threshold-independent manner. The results of this analysis are displayed in Fig. 4 and Supplementary Table 4. Each of the four mirZIPs led to a positive enrichment score for the SSM-UP subtype signature, confirming the ‘anti-SSM' activity of all four microRNAs. Moreover, while most functions were specifically modulated by single microRNAs, upregulation of genes involved in ‘TNF signalling pathway via NFkB' was promoted by downregulation of three microRNAs (miR-194, miR-200b and miR-429). Interestingly, downregulation of miR-203 on one side induced genes of the ‘TGF-β pathway via SMAD activation', and on the other repressed cell cycle genes (‘E2F Targets' and ‘G2M checkpoint'). It is therefore likely that miR-203 indirectly promotes cell cycle by downregulating TGF-β pathway genes. ‘MYC targets' and EMT markers (‘Epithelial Mesenchymal Transition' and ‘NABA ECM regulators') were upregulated on silencing of, respectively, miR-194 and miR-200b. miR-429 is likely to have an immunosuppressive function, since its downregulation was found to promote expression of genes involved in inflammation (‘Interferon Gamma Response' and ‘Inflammatory Response').

### Identification of core microRNA predicted targets

**Figure 5:**
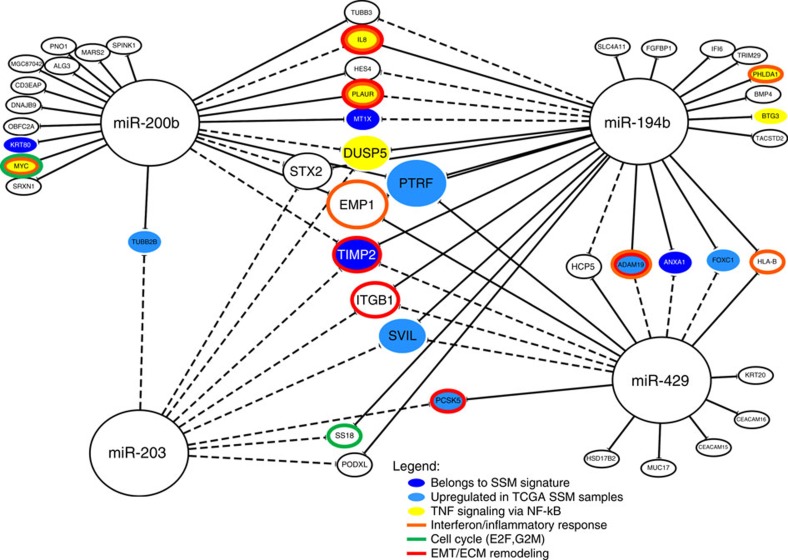
microRNAs antagonizing the SSM phenotype share mRNA targets. Network of interactions between the four functionally validated microRNAs and their core target mRNAs. The network reports mRNA–microRNA interactions detected both *in vitro* and *in vivo* (solid lines) and those detected only *in vivo* (dashed lines). The mRNA node size is proportional to the number of microRNAs with which it is linked and to the number of solid links. A colour code is used to highlight genes involved in relevant pathways or signatures.

To single out potential mediators of the anti-SSM action of the four validated microRNAs, we defined a set of key genes (‘core targets') associated with each microRNA according to both *in vitro* and *in vivo* observations. In particular, a core target of a microRNA should: (i) be significantly *in vitro* upregulated on silencing of a given microRNA, as described above; (ii) have a negative association coefficient with the same microRNA expression in human CRC samples, as determined by SLR analysis in the TCGA expression data set, considering each selected gene as response variable and the four functionally validated microRNAs as explanatory variables. According to this procedure, 22 core targets were found for miR-194, 18 for miR-200b and 10 for miR-429. No core targets were found for miR-203, likely because the significantly modulated genes *in vitro* by its downregulation were only three. However, SLR analysis, carried out on all four microRNAs for all *in vitro* upregulated genes, highlighted negative association with miR-203 for eight core targets of other microRNAs. A similar cross-association was also observed between the other microRNAs. In total, 21 genes were associated to more than 1 microRNA, with 2 genes (*EMP1* and *PTRF*) being core targets of 3 microRNAs. Figure 5 displays the whole network of microRNA–mRNA interactions, with colour codes depicting involvement in the SSM subtype and/or functional pathways. The abundant cases of multiple gene/microRNA connections can be explained by the fact that all four microRNAs are predicted to control the same phenotypic subdivision (SSM versus non-SSM). Of note is that miR-194, miR-200b core-targets seem to be more strongly involved in the TNF pathway regulation. Moreover genes belonging to SSM signature or at least strongly upregulated in the SSM subtype are among those genes with an higher degree in the network (2–3), showing a combined regulation of the SSM associated genes by all the four microRNAs.

## Discussion

The wide molecular and clinical heterogeneity of CRC-prompts research aimed at defining more homogeneous subtypes of the disease. Among the possible ways to achieve this goal, definition of subtypes based on distinctive transcriptional profiles recently emerged as a powerful approach^1^,^2^,^3^. However, no mechanistic explanation has been provided to justify the different transcriptional make up of the various subtypes. In this work, we explored the possible role of microRNAs, by developing and applying an analysis pipeline, MMRA, aimed at identifying microRNAs with a potential ‘master regulator' role.

The MMRA pipeline has multiple innovative features. It works on large data sets of paired mRNA–microRNA expression, in which samples are subdivided in two or more subgroups based on mRNA signatures. It integrates statistics and network theory in two serially combined analysis modules that, in principle, could also be used independently. However, the network analysis module, that we found to improve accuracy of the analysis, could not be employed in the absence of the microRNA filtering provided by the statistics module, due to excessive computational demand. MMRA also makes an original use of the well-known ARACNE algorithm, employing MI to reconstruct mixed mRNA–microRNA regulatory networks, the ‘regulons'. Indeed, existing tools use MI for integrative analysis of microRNA-targets expression profiles^22^,^23^, but they do not use ARACNE for network reconstruction. In another case, a pipeline built to identify microRNA-transcription factor networks in Glioblastoma includes ARACNE, but only to define gene–gene interactions, while microRNA–gene interactions are identified through TargetScan^24^. Therefore ARACNE has never been applied to mixed mRNA–microRNA data as in MMRA. The above features and findings establish the MMRA pipeline as a first-in class tool to successfully combine multiple computational approaches to find driver microRNAs within paired mRNA–microRNA expression data sets.

MMRA was applied to a large paired mRNA–microRNA data set of CRC samples and highlighted the involvement of candidate microRNAs in regulating CRC subtypes. Notably, most candidates were predicted to downregulate genes of the poor prognosis SSM subtype. *In vitro* functional validation experiments confirmed the reliability of the pipeline and the role of miR-194, miR-200b, miR-429 and miR-203 in the negative regulation of this subtype. Interestingly, these miRNAs inhibit metastasis through negative regulation of two key biological properties: EMT and stemness. In fact, several lines of evidence suggest a connection between metastasis induction and stem-like properties acquisition from cancer cells undergoing EMT^25^,^26^,^27^. The same link between EMT and stemness is observed for the four identified microRNAs: they all are inhibitors of the cancer stem cell phenotype^26^,^28^ and are EMT repressors. In particular miR-200b and miR-429, both part of miR-200 family, are involved in a feedback regulatory loop with zinc finger E-box-binding factors *ZEB1* and *ZEB2*, which ensures a switch-like regulation of EMT^27^,^29^. Moreover, miR-194 and miR-203 are repressed by *ZEB1* (refs 26, 30). Involvement of the four microRNAs in EMT was also confirmed by the GSEA analysis applied to identify signalling pathways modulated in cell lines on silencing of each microRNA by its respective mirZIP. This analysis highlighted a consistent and possibly cooperative effect of all four microRNAs in modulating multiple EMT pathways: TNF via NFkB signalling, TGF-β pathway, EMT marker genes and MYC targets. TNF signalling via NFkB is required in cancer cells to maintain a mesenchymal phenotype^31^,^32^,^33^,^34^. Involvement of miR-203 in the regulation of TGF-β pathway was already experimentally observed^35^ and the role of TGF-β pathway in EMT is well known^36^,^37^,^38^. Finally, *MYC* is involved not only in EMT but also in cell pluripotency acquisition working as a connection between stemness and EMT^39^,^40^,^41^. In particular, *MYC* plays a dominant role in regulating several miRNAs in the reprogramming process to stemness penothype^41^,^42^. Our functional validation highlighted regulation of *c-MYC* and its targets on silencing of miR-194, not previously reported as a *MYC*-regulated microRNA. Cooperation of the above microRNAs in driving a non-SSM phenotype is further substantiated by the finding of a consistent fraction of shared core mRNA targets, as illustrated in Fig. 5.

We recently showed that a large fraction of genes overexpressed in SSM samples of CRC are indeed expressed by stromal rather than cancer cells^6^. This poses the question of whether the microRNA–mRNA associations identified by MMRA, where the target mRNAs are upregulated in SSM samples, reflects tumour–stroma interactions rather than cancer cell-intrinsic regulatory circuits. We therefore exploited our previous analyses^6^ to verify whether the identified core target genes are expressed by stromal cells. Estimate of stromal contribution was available for 38 of the 45 identified core targets. Interestingly, none of them had an estimated stromal contribution above 50%, only 9 of 25–50% and 29 had <25% estimated stromal contribution. These result confirm the power of an analysis based on microRNA–mRNA interactions detected both *in vitro* and *in vivo* to highlight regulatory circuits mostly occurring in cancer cells.

Of particular importance is therefore the *in vitro* validation of the driver role of of miR-194, miR-200b, miR-429 and miR-203 in bringing CRC cells away from the SSM state. Despite the change of sampling material (cell lines versus human CRC tissue) and the limited number of cell lines available for the paired mRNA–microRNA analysis, the negative relationship between expression of these microRNAs and SSM subtype was confirmed. Moreover, experimental downregulation of these microRNAs caused a detectable shift of the CRC cell mRNA transcriptome towards the SSM state, even though only one microRNA at a time was silenced. These results show that the integrative approach combining supervised statistics with unsupervised network analysis, at the basis of our MMRA pipeline, allowed reliable detection of microRNAs with a driving role in determining molecular and biological features of CRC.

## Methods

### Pre-processing TCGA microRNA expression data set assembly

To generate a matched mRNA–microRNA expression data set of primary CRC, we started from our previously assembled 450-sample TCGA mRNA data set^6^, available as ExperimentData package from Bioconductor: http://www.bioconductor.org/packages/release/data/experiment/html/TCGAcrcmRNA.html. For all these samples, in April 2013 we downloaded from the TCGA data portal (https://tcga-data.nci.nih.gov/tcga/) level 3 microRNA expression data generated by small RNA sequencing corresponding to the ‘microRNA.txt' file. Level 3 small RNAseq data are preprocessed by TCGA as described^43^. Indeed, data processing methods alternative to those employed by TCGA could provide different results, as discussed by Dillies *et al.*^44^, but this would require direct access to sequence reads. Downloaded data were initially assembled into two matrices, one for the ‘GA' platform (229 samples) and one for the ‘Hiseq' platform (221 samples). No sample was profiled through both platforms, but the two data sets had an identical distribution. We therefore filtered out those microRNAs having a s.d. equal to zero (that is, not detected) and those with an absolute spearman correlation with the GA versus Hiseq platform >0.65 (90th percentile of the distribution). Finally, we combined the two microRNA data sets into a unique set providing expression values for 434 microRNAs in 337 colon and 113 rectal adenocarcinomas. The data set is available as ExperimentData package from Bioconductor:

http://www.bioconductor.org/packages/release/data/experiment/html/TCGAcrcmiRNA.html. Classification of the TCGA samples in transcriptional subtypes according to the CCS, CRCA and CCMS classifiers were obtained from Supplementary Table 1 of Isella *et al.*^6^ At the end of this processing step, we had all the necessary data for the MMRA pipeline: (i) paired mRNA–microRNA expression data; (ii) samples subdivision by transcriptional classifiers.

### Availability of the MMRA pipeline

MMRA is available at http://eda.polito.it/MMRA/, It is subdivided into four major steps described in the following sections.

### MMRA step 1–microRNAs differential expression analysis

The aim of the first step was to identify microRNAs with subtype-specific expression. To perform differential microRNA expression analysis, we organized samples according to their available mRNA-based classification^6^. Then we defined ‘subtype core' samples by restricting subtype membership to those samples that, according to NTP, in addition of having FDR <5% (standard threshold for the NTP algorithm), also had a distance from the nearest template (*δ*) <0.8. This value corresponds to the 95th percentile of the distribution of the distances of all samples from all centroids. The distance threshold was added to strictly select those samples that are strongly associated to the class, avoiding the introduction of noise in the differential expression analysis. The number of core samples defined with the above procedure is the following: CCS (150, 67, 87), CCMS (18, 54, 42, 82, 65, 54) and CRCA (50, 40, 42, 94, 70). Although not perfectly balanced, the size of each subtype core remains comparable. In the analysis, a subtype-specific microRNA should have significant differential expression between core samples of a given subtype and all other samples, excluding from the analysis those samples assigned to the test subtype but with low confidence. Differential expression analysis was performed through a Kolmogorov–Smirnov test, including a fold-change (FC) threshold. Kolmogorov–Smirnov test was chosen because it does not assume a priori any data distribution and its use for differential expression analysis is well documented^45^,^46^. To address the possible issue of sample size, we took advantage of a Kolmogorov–Smirnov test with bootstrapping (function ks.boot implemented in the R package ‘Matching'^47^). A microRNA was considered differentially expressed in a subtype if the Kolmogorov–Smirnov *P* value was <0.001 and the absolute FC was >2. The adequacy of the selected thresholds was assessed by a permutation-based estimate of the FDR, that is, the estimated percentage of microRNAs identified by chance. For each pair of chosen Kolmogorov–Smirnov *P* value and FC thresholds, the FDR was computed reshuffling 1,000 times the samples constituting the microRNA data set. The mean value of microRNAs significantly differentially expressed in these 1,000 experiments was computed and then compared with the number of microRNAs differentially expressed in our step of the pipeline. As shown in Supplementary Table 5, the FDR obtained for the selected pair of thresholds (*P* value 0.001, FC >2) was the minimum among all tested combinations in CCS and CRCA classifiers. For what concerns CCMS, as can be observed from Supplementary Table 5, the global FDR initially decreases from 6 to 3% and then to around 1%, finally stabilizing around 0.8–0.6%. It follows that, according to the global FDR value, all the three combinations ‘0.001–1.5 × ', ‘0.001–2 × ' and ‘0.001–2.5 × ' can be in principle considered very good (FDR <1%). It should be considered that microRNA differential expression analysis corresponds to the first step of the MMRA pipeline, therefore the threshold combination should be sufficiently selective but at the same time not too stringent. For this reason, of the three tests with FDR <1% we considered the intermediate option ‘0.001–2 × ', corresponding to 54 microRNAs, as the preferred choice. The other two threshold choices would lead to 24 or 143 microRNAs over 475, possibly too stringent or not sufficiently selective. We therefore checked what would change in the results of MMRA using for differential expression the combination ‘0.001–2.5 × ' corresponding to the minimum FDR. The ‘0.001–2.5 × ' combination yields only four microRNAs at the end of the pipeline, of which only one is confirmed in cell lines (25% versus 38% in our chosen test). Importantly, this configuration would miss miR-194 and miR-429, that were functionally validated by loss-of-function in cell lines. We also verified the possible changes in the list of differentially expressed miRs without using the distance from the class centroid to select subtype core samples. The results confirmed our hypothesis: despite the presence of more samples per subtype, the resulting set of differentially expressed microRNAs was smaller, highlighting greater within-subtype heterogeneity. Moreover, the resulting microRNAs were all contained in the previous list based on subtype cores.

### MMRA step 2–target transcripts enrichment analysis

In the second MMRA step, for each microRNA differentially expressed in a given CRC subtype, we performed a target enrichment analysis in the gene signature corresponding to the CRC subtype in which the microRNA was differentially expressed. MicroRNA's target transcripts were predicted following the procedure discussed in Riba *et al.*^48^ More precisely, we combined the results of four prediction databases (miRTarBase 2.5 (ref. 49), doRiNA-PicTar 2012 (ref. 50), microRNA.org 2010 (ref. 51), PITA 2007 (ref. 52) and TargetScan 6.1 (ref. 53)), requiring the agreement of at least two of them to include a putative target in our analysis. For each database we always chose the most stringent option among those proposed by the database. Then we added to this list all the experimentally validated targets contained in the miRTarBase 2.5 database. Next, to perform target enrichment analysis and all the further pipeline steps, the gene signatures CCS, CRCA and CCMS were downloaded from the Supplementary Materials of the works^1^,^2^,^3^. Then the genes of the three classifiers were organized as follows. For each of the CRCA and CCS subtypes, we defined two gene signatures: the first, that we called ‘UP', containing all the genes with prediction analysis of microarray values >0, and the second, ‘DOWN', containing all the genes with prediction analysis of microarray value <0, as reported in the Supplementary Tables of the works. For each CCMS subtype we selected, as UP genes, those with log2 FC >0.5 and adjusted *P* value <0.05, and as DOWN genes those with log2 FC <−0.5 and adjusted *P* value <0.05. The FC and *P* value thresholds are the same as originally used by the authors^3^. For each microRNA identified in step (i) as differentially expressed in a given CRC subtype, to evaluate an enrichment of predicted targets in the UP or DOWN signature of that subtype, we calculated a Bonferroni-adjusted Hypergeometric test *P* value and the observed/expected (O/E) ratio. To choose optimal *P* value and O/E ratio thresholds, we implemented a FDR computation as follows. For each subtype, a random set of microRNAs, of the same size of the subtype-specific microRNA set, is selected and tested for target enrichment in the UP or DOWN signatures of the same subtype, according to a given combination of *P* value and O/E ratio thresholds. After 1,000 random iterations, the mean number of randomly significant microRNAs across a classifier is compared with the number of ‘true' significant microRNAs for the same classifier. The test is performed for a list of *P* value and O/E ratio thresholds, and finally the threshold combination that minimizes FDR is chosen. This FDR analysis is also included in the MMRA pipeline available online. Interestingly this FDR computation controls also possible biases due to the presence of databases containing larger target lists. In fact, if this kind of bias exists, it will also affect the random null model. Therefore, thresholds that minimize the FDR also minimize the possible bias consequences. The FDR values obtained for each classifier and for each threshold combination are reported in Supplementary Table 6. Indeed, the three classifiers required different thresholds to minimize FDR. *P* value thresholds: 0.001 for CRCA, 0.01 for CCMS and 0.001 for CCS; O/E thresholds: 1.5 for CRCA, 1.5 for CCMS and 2.5 for CCS.

### MMRA step 3–network analysis

Network analysis was performed using the ARACNE information-theoretic algorithm for inferring transcriptional interactions^11^. The software was downloaded (http://wiki.c2b2.columbia.edu/califanolab/index.php/Software/ARACNE) and included in the pipeline to infer interactions between each microRNA selected by the previous steps and any mRNA from the paired data set. Indeed, ARACNE is typically employed to reconstruct extended networks with more than one ‘marker' hub, while in our analysis, having only one hub, we could in principle apply a simple miRNA-gene MI-based analysis. However, such analysis would yield links between the microRNA and all the expressed genes. An important issue would then be how to filter the links, possibly in a more refined way than just by MI thresholding. We solved this problem using the two main filtering procedures implemented in the ARACNE algorithm: (i) estimate of MI significance trough the reconstruction of a null model by sample reshuffling independently for each row (gene), and (ii) bootstrapping procedure, with random exclusion of a subset of samples, to generate a consensus network including edges supported across many bootstrap networks. Edge support significance is then estimated by randomly shuffling the edge positions, to create a null model of network consensus. For each microRNA selected at the previous steps, data preparation for ARACNE involved the setting up of an expression matrix (X) row-wise combining the entire mRNA expression TCGA data set with the expression values of the single microRNA under analysis. To generate a matrix compatible with the standard ARACNE pre-processing steps, we inverted log2 transformation of the expression data set: naming Xij the elements of the above-described expression matrix, we obtained the called ‘linear expression matrix' Y through the following operation Yij=2^Xij^. Then, standard ARACNE pre-processing involves quantile normalization of the data set Y, log2 transformation and filtering of those genes with a s.d. <1.2. For MMRA the only edges of interest are those connecting the microRNA to mRNAs, therefore the algorithm is run imposing the microRNA as the only hub of the network. The chosen MI *P* value significance threshold (10^−7^) and bootstrapping *P* value threshold (10^−12^ after 100 bootstrapped networks) are the originally recommended ones^54^. Subsequently, each of the consensus networks constructed around the selected microRNAs (the ‘regulons'), is tested for significant enrichment in subtype signature genes respect to a random null model. To this end the master regulator analysis (MRA) algorithm is used, as previously described^12^,^18^, to evaluate the statistical significance (*P* values computed by Fisher's exact test) of the overlap between the ‘regulon' of each microRNA and the gene signature of the subtype in which the microRNA was identified as differentially expressed at the previous steps. To assess the sensitivity and specificity of our approach, we compared our results with a null model constituted of the networks centred in microRNAs that were expressed (detected in more than 45 of the 450 samples) but not differential in any subtype of any classifier (signal-to-noise ratio, corresponding to FC divided by within-group standard deviation, <−0.05). The regulons of the microRNAs constituting the null model were also required to have an intersection with any regulon of the previously selected candidate microRNAs <70%. We obtained, in this way, a null model constituted of nine microRNAs and their regulons. Then, we estimated the threshold for the MRA *P* value comparing the *P* values obtained in our analysis with those of the null model. In detail, we performed MRA also on the nine null model regulons, testing the enrichment in signature genes of all the CRC subtypes. Then, from the *P* value distribution of the null model we chose a threshold for the MRA of *P*=10^−4^, corresponding to the 95th percentile of the null model. At the end of this step all the microRNAs having a MRA *P* value in their associated subtype >10^−4^ were filtered out. To test, for some microRNAs, if signature genes were not only enriched in the microRNA regulons but also, within the regulons, were among those with the highest MI content we used Preranked GSEA^21^. mRNAs contained in the regulon were ranked according to their MI values. Then with Preranked GSEA we tested if signature genes where significantly associated to high/low values of MI or they were randomly distributed.

### MMRA step 4–SLR analysis

In this step, to filter out weak microRNA–mRNA relations within the regulons, MMRA employs SLR, a procedure previously adopted for transcription factor/target analysis^12^,^18^. The assumption at the basis of SLR application in such case was that the logarithm of a target mRNA expression level is a linear function of the logarithm of the expression level of its putative transcription factor regulator(s). We considered that such first-order approximation is widely used also to model mRNA–microRNA interactions (see for instance ref. 55); therefore SLR could also be applied in a case where the regulators are microRNAs. SLR was performed employing together all the microRNAs selected across the previous steps, against all gene signatures of all three classifiers, without making any distinction between microRNAs identified for one or another classifier. The SLR procedure involved the construction of a linear model for each signature gene, as follows: the log2-expression level of the gene was considered the response variable, and the log2-expression levels of microRNAs linked by ARACNE to the gene were considered as the explanatory variables. Then a stepwise algorithm is used to select the best minimal set of explanatory variables within the model. Akaike information criterion was used as the stop criterion. The output of SLR was reorganized at the microRNA level, to include, for each microRNA, a list of response variables (subtype signature genes associated by ARACNE) to which it was associated by SLR. The extent of modulation of a given subtype by a given microRNA can then be estimated as the fraction of signature genes for that subtype (UP or DOWN) whose expression is approximated by the microRNA according to SLR analysis (positive or negative coefficient). To estimate a significance threshold for this step we considered the distribution of the results for all the selected microRNAs in all the CRC subtypes. These results are expected to include a small subset of true associations, also selected across the previous steps, and a larger set of random associations. We therefore selected the 90th percentile of the fraction values, corresponding to a threshold of 13% of associated signature genes. To generate the final output of the MMRA pipeline, significant fractions of associated subtype genes are provided only for the microRNA-subtype associations also selected in the previous steps.

### Paired cell line expression data assembly and classification

For 18 CRC cell lines, obtained as described^19^, microRNA expression profiling was obtained by Illumina TruSeq Small RNA sequencing on the Illumina HiSeq 2000 platform. All cell lines were maintained in their original culturing conditions according with supplier guidelines. Cells were ordinarily supplemented with FBS at different concentrations, 2 mM L-glutamine, antibiotics (100 U ml^−1^ penicillin and 100 mg ml^−1^ streptomycin) and grown in a 13 37 °C and 5% CO_2_ air incubator. Cells were routinely screened for absence of mycoplasma contamination using the Venor GeM Classic kit (Minerva biolabs). The identity of each cell line was checked by Cell ID System and by Gene Print 10 System (Promega), throught short tandem repeats (STR) at 10 different loci (D5S818, D13S317, D7S820, D16S539, D21S11, vWA, TH01, TPOX, CSF1PO and amelogenin). Amplicons from multiplex PCRs were separated by capillary electrophoresis (3730 DNA Analyzer, Applied Biosystems) and analysed using GeneMapperID software from Life Technologies. Resulting cell line STR profiles were cross-compared and matched with the available STR repositories online databases. Raw data were analysed by Genomatix according to its standard pipeline (‘myGenomatics'; www.genomatix.de). Read counts provided by Genomatix were then normalized with Deseq^56^, which is widely used and has overall good performances^44^. For the same cell lines, normalized global mRNA expression profiles were extracted from the 151-cell lines data set^19^ available at Gene expression Omnibus (data set GSE59857, samples: GSM1448073, GSM1448118, GSM1448124, GSM1448132, GSM1448134, GSM1448142, GSM1448143, GSM1448146, GSM1448147, GSM1448164, GSM1448175, GSM1448176, GSM1448177, GSM1448179, GSM1448180, GSM1448194, GSM1448195 and GSM1448212).

The joint mRNA–microRNA data set for these 18 CRC cell lines is available in Bioconductor (http://www.bioconductor.org/packages/release/data/experiment/html/CRCL18.html). The 18 cell line mRNA classification in molecular subtypes was downloaded from Supplementary Table 2 in ref. 19, so that differential microRNA expression across subtypes could be assessed also in cell lines.

### Consolidation of microRNA-subtype associations

The consolidation was based on two main criteria: (i) differential expression between cell lines of the target subtype and other cell lines, with the same direction as found in TCGA data analysis. In particular, the following FC thresholds were used: 1.321722 for CCMS, 1.237681 for CRCA and 1.521026 for CCS. These thresholds were chosen based on a null model obtained performing the same analysis on 1,000 random sets of microRNAs of the same size of the MMRA output (in this case *n*=24), ad selecting the 90th percentile of the FC distribution obtained in this null model; (ii) Fraction of subtype signature genes whose expression is significantly correlated to the miRNA across subtypes (absolute Spearman *r*>0.9 and 0.829, respectively, for CRCA and CCMS, corresponding to a *P* value <0.1; this analysis could not be done for CCS due to poor reliability of correlation estimates across only three subtypes). The fraction of signature genes with an expression pattern significantly correlated to that of the microRNA that was considered significant is 5% in CCMS and 10% in CRCA. Also these thresholds were estimated through a null model constructed from 1,000 random sets of microRNAs of the same size of those passing step 1, and calculating the 90th percentile of the distribution of the percentage of signature genes correlated with the microRNA.

### Selection of microRNAs for functional validation

To prioritize microRNAs on which to perform the functional validation, we added two criteria: (i) microRNAs identified by the MMRA pipeline in more than one classifier; (ii) correlation analysis as described in point (ii) above, but considering only the microRNA targets in the signature and not all signature genes. We then selected those microRNAs with a fraction of correlated genes higher in this analysis with respect to the one performed considering all the genes of the signature. In total, four microRNAs optimally fulfilled these criteria and were selected for further analysis: miR-194, miR-200b, miR-203 and miR-429. Interestingly, all four microRNAs are downregulated in the SSM subtype, and their targets within the SSM signature are upregulated.

### Selection of cell lines for functional validation

All four microRNAs selected for functional validation had a higher expression in non-SSM CRC cell lines. In principle, if they have a driver role, their downregulation in such cells should make the transcriptome shift towards the stem subtype. We therefore selected, among the 18 CRC cell lines, those non-SSM in which at least one of the microRNAs was expressed at higher levels than in SSM cell lines (log2 ratio >0). In addition, candidate cell lines should also express, respect to SSM cells, lower levels of genes belonging to the ‘SSM-UP' signature (average log2 ratio <0). Such downregulation should be further enhanced when considering microRNA target genes within the signature. One cell line, NCIH508, was found to fulfil such criteria for all four microRNAs. Cell line HT29 satisfied these criteria for three microRNAs (miR-194, miR-200b and miR-429) and SW403 was selected for miR-429.

### Transcriptional response to microRNA silencing experiments

Details about cell transductions with microRNA-targeting constructs, and the expression profiles obtained from transduced cells, are available in the GEO data set GSE59883. To functionally verify whether the four microRNAs identified by MMRA modulate the SSM phenotype, we assembled a ‘SSM-UP signature' joining the CCMS4-UP, CRCA5-UP and CCS3-UP signatures. Similarly, a ‘SSM-DOWN' signature was obtained joining the CCMS4-DOWN and CRCA5-DOWN signatures (CCS has no CCS3-DOWN genes). Genes with differential expression between mirZIP-transduced and scramble cells were identified through a combined FC and Student's *t*-test analysis (absolute FC >1.5 and *t*-test *P* value <0.05). To identify genes whose differential expression was specifically due to microRNA downregulation, we filtered out those genes satisfying the same criteria also between wild-type and scramble-transduced cells. To test for enrichment of stem signature genes among genes upregulated by mirZIP transduction, we performed Hypergeometric test with a standard significance threshold of *P*<0.05. To verify whether cells change of subtype after microRNA silencing, we assembled a data set composed of the 18 original CRC cell lines (non-normalized data from the above-described GSE59857, samples) plus the mirZIP, scramble and WT lines (non-normalized expression profiles from GSE59883, all samples). The obtained data set was Loess normalized. Then to test whether cell lines change phenotype after microRNA silencing, we applied NTP classification, using the SSM-UP and SSM-DOWN signatures as centroids, to a series of data sets each composed of the 18 CRC lines plus one mirZIP-transduced duplicate (averaged) at a time. Addition to the 18-panel of one single cell at a time was chosen to minimize distortions during the data standardization phase of NTP.

## Additional information

**Accession codes:** The novel microarray data set of cells transduced with miRNA inhibitors is deposited at GEO under the accession code GSE59883. The miRNA expression data for the CRC cell lines is deposited at Bioconductor under the accession code CRCL18.

**How to cite this article:** Cantini, L. *et al.* MicroRNA–mRNA interactions underlying colorectal cancer molecular subtypes. *Nat. Commun.* 6:8878 doi: 10.1038/ncomms9878 (2015).

## Supplementary Material

Supplementary Information Supplementary Figures 1-2, Supplementary Tables 1-6, Supplementary Note and Supplementary References.

Supplementary Data 1 List of microRNAs differentially expressed in each subtype. For each subtype of each classfier, the table reports differential microRNAs and whether they are up-regulated or down-regulated.

Supplementary Data 2 A double filter for significant regulation, based on t-test (p< 0.05) and fold-change (+/- 1.5x), was applied to the 14345 genes expressed in HT-29 cells, to identify mRNAs modulated by the various microRNA silencers. The total stem genes mapped on this dataset were 185 and 230, respectively Up and Down in the Stem subtype (See online methods for further details).

Supplementary Data 3 A double filter for significant regulation, based on t-test (p<0.05) and fold-change (+/-1.5x), was applied to the 14345 genes expressed in HT-29 cells, to identify mRNAs modulated by the various microRNA silencers. The total stem genes mapped on this dataset were 122 and 246, respectively Up and Down in the Stem subtype (See online methods for further details).

Supplementary Data 4 A double filter for significant regulation, based on t-test (p< 0.05) and fold-change (+/-1.5x), was applied to the 14927 genes expressed in SW403 cells, to identify mRNAs modulated by the various microRNA silencers. The total stem genes mapped on this dataset were 114 and 245 , respectively Up and Down in the Stem subtype (See online methods for further details).

## Figures and Tables

**Figure 1:**
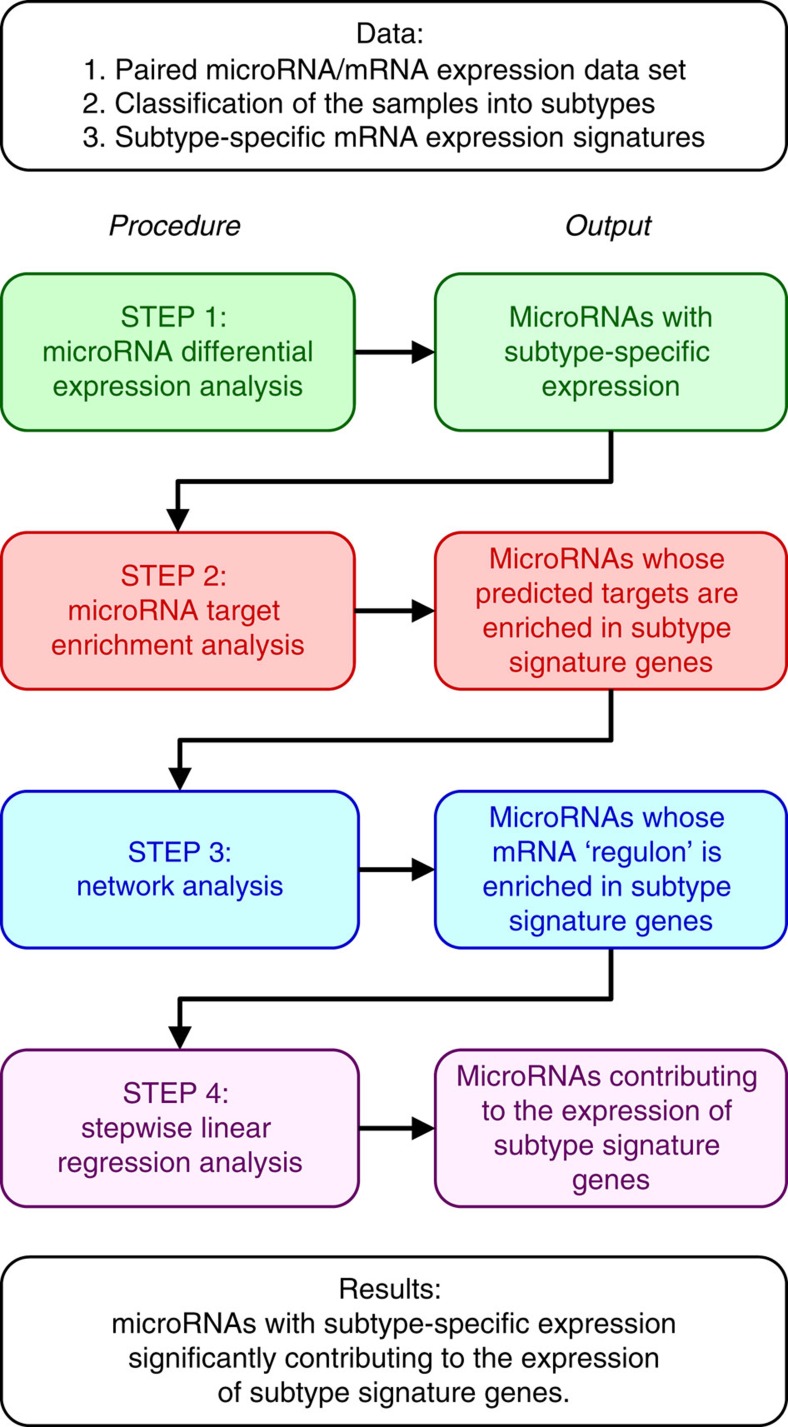
Schematic representation of the MMRA workflow. The schema reports the data required as initial input, the four analytic steps with the respective outputs, and the final output of the pipeline.

**Table 1:** MicroRNAs identified by MMRA with differential expression across CRC subtypes and associated to subtype-specific mRNA signatures.

**MicroRNA**	**MicroRNA expression**	**Associated subtype signature**	**SLR-estimated association (%)**	**Other associated subtype signatures**
hsa-miR-223	Up in CRCA1	Down in CRCA1	15	CRCA-UP4
hsa-miR-181d	Down in CRCA1	Up in CRCA1	21	CRCA-DN4
hsa-miR-375	Up in CRCA2	Down in CRCA2	14	—
hsa-miR-103	Down in CRC5	Up in CRCA5	30	CRCA-DN2+DN3+DN4
hsa-miR-130b	Down in CRCA5	Up in CRCA5	37	CRCA-UP1+DN2+DN3+DN4
hsa-miR-135b	Down in CRCA5	Up in CRCA5	14	—
hsa-miR-141	Down in CRCA5	Up in CRC5	24	CRCA-DN2+DN3
hsa-miR-143	Up in CRCA5	Up in CRC5	14	CRCA-DN2
hsa-miR-148a	Down in CRCA5	Up in CRCA5	30	CRCA-DN2+DN3+DN4
hsa-miR-153	Down in CRCA5	Up in CRCA5	19	CRCA-DN2+DN3
hsa-miR-17	Down in CRCA5	Up in CRCA5	24	CRCA-DN2+DN3+DN4
hsa-miR-194	Down in CRCA5	Up in CRCA5	16	CRCA-DN2
hsa-miR-19b	Down in CRCA5	Up in CRCA5	20	CRCA-DN2+DN4
hsa-miR-200b	Down in CRCA5	Up in CRCA5	24	CRCA-DN2+DN3
hsa-miR-203	Down in CRCA5	Up in CRCA5	21	CRCA-DN2
hsa-miR-20a	Down in CRCA5	Up in CRCA5	29	CRCA-UP1+DN2+DN4
hsa-miR-429	Down in CRCA5	Up in CRCA5	20	CRCA-DN2
hsa-miR-33a	Down in CRCA5	Up in CRCA5	24	CRCA-DN2+DN4
hsa-miR-218	Up in CRCA5	Up in CRCA5	25	CRCA-DN2+DN3+DN4
hsa-miR-141	Down in CCS3	Up in CCS3	31	CCS-DN1
hsa-miR-200a	Down in CCS3	Up in CCS3	15	—
hsa-miR-501	Up in CCMS1	Down in CCMS1	18	CCMS-DN3+UP4
hsa-miR-141	Down in CCMS4	Up in CCMS4	21	CCMS-DN1+DN3
hsa-miR-148a	Down in CCMS4	Up in CCMS4	24	CCMS-DN1+DN3+UP5
hsa-miR-153	Down in CCMS4	Up in CCMS4	15	CCMS-DN1+DN3
hsa-miR-200a	Down in CCMS4	Up in CCMS4	15	CCMS-DN1+DN3
hsa-miR-33a	Down in CCMS4	Up in CCMS4	23	CCMS-DN1+DN3
hsa-miR-130b	Down in CCMS4	Up in CCMS4	28	CCMS-DN1+DN3+UP5
hsa-miR-194	Down in CCMS4	Up in CCMS4	14	—
hsa-miR-362-3p	Down in CCMS4	Up in CCMS4	28	CCMS-DN1+DN3+UP5
hsa-miR-429	Down in CCMS4	Up in CCMS4	15	CCMS-DN1+DN3
hsa-miR-203	Down in CCMS4	Up in CCMS4	20	CCMS-DN1+DN3
hsa-let-7c	Up in CCMS4	Up in CCMS4	22	CCMS-DN1+DN3
hsa-miR-1-2	Up in CCMS4	Up in CCMS4	14	CCMS-DN1+DN3

The table reports the MMRA pipeline output. The first column reports the identified microRNAs, the second column the subtype in which the microRNA is differentially expressed and if it is upregulated or downregulated. The third column reports the gene signature associated to each microRNA. The fourth column reports the percentage of signature genes whose expression is recapitulated by the microRNA expression in SLR analysis. The rightmost column reports other subtype signatures of the same classifier associated with the microRNA by SLR analysis.

**Table 2:** MicroRNA downregulation in CRC cell lines leads to modulation of SSM subtype genes and change in subtype assignment.

**Cell line**	**HT29**			**NCIH508**				**SW403**
**Targeted microRNA**	**mir-194**	**mir-200b**	**mir-429**	**mir-194**	**mir-200b**	**mir-429**	**mir-203**	**mir-429**
Upregulated genes (total)	252	567	163	20	6	20	32	104
Fold enrichment in SSM genes	5.23	3.01	2.85	5.9	19.7	59.2	33.3	7.55
Enrichment *P* value	2.4E-08	2.9E-06	1.4E-02	0.14	0.049	2.2E-16	3.8E-12	0.0001
Downnregulated genes (total)	244	411	115	6	1	2	11	83
Fold enrichment in non-SSM genes	6.65	1.67	0.54	8.8	0	5.9	1.8	4.4
Enrichment *P* value	1.6E-14	0.034	0.29	9.3E-05	0.983	2.9E-04	0.158	0.002
Original subtype	SSM			Non-SSM				Non-SSM
Original FDR	0.88			0.28				0.002
New subtype	SSM	SSM	SSM	SSM	SSM	SSM	SSM	Non-SSM
New FDR	0.200	0.001	0.005	0.004	0.005	0.004	0.002	0.002

The table reports, for each cell line and each targeted microRNA, the total number of genes upregulated and downregulated after microRNA silencing, the enrichment of SSM genes among upregulated genes and of non-SSM genes among downregulated genes, and classification of the cell line into SSM or non-SSM subtype before and after microRNA silencing with the respective classification confidence expressed as FDR.
