# Supplementary material for: MicroRNA–mRNA interactions underlying colorectal cancer molecular subtypes
Source: Nat Commun. 2015 Nov 17;6:8878. doi: 10.1038/ncomms9878 (PMC4660217; doi:10.1038/ncomms9878)
Supplement: Supplementary Information — Supplementary Figures 1-2, Supplementary Tables 1-6, Supplementary Note and Supplementary References. [file ncomms9878-s1.pdf]

Supplementary Figure 1

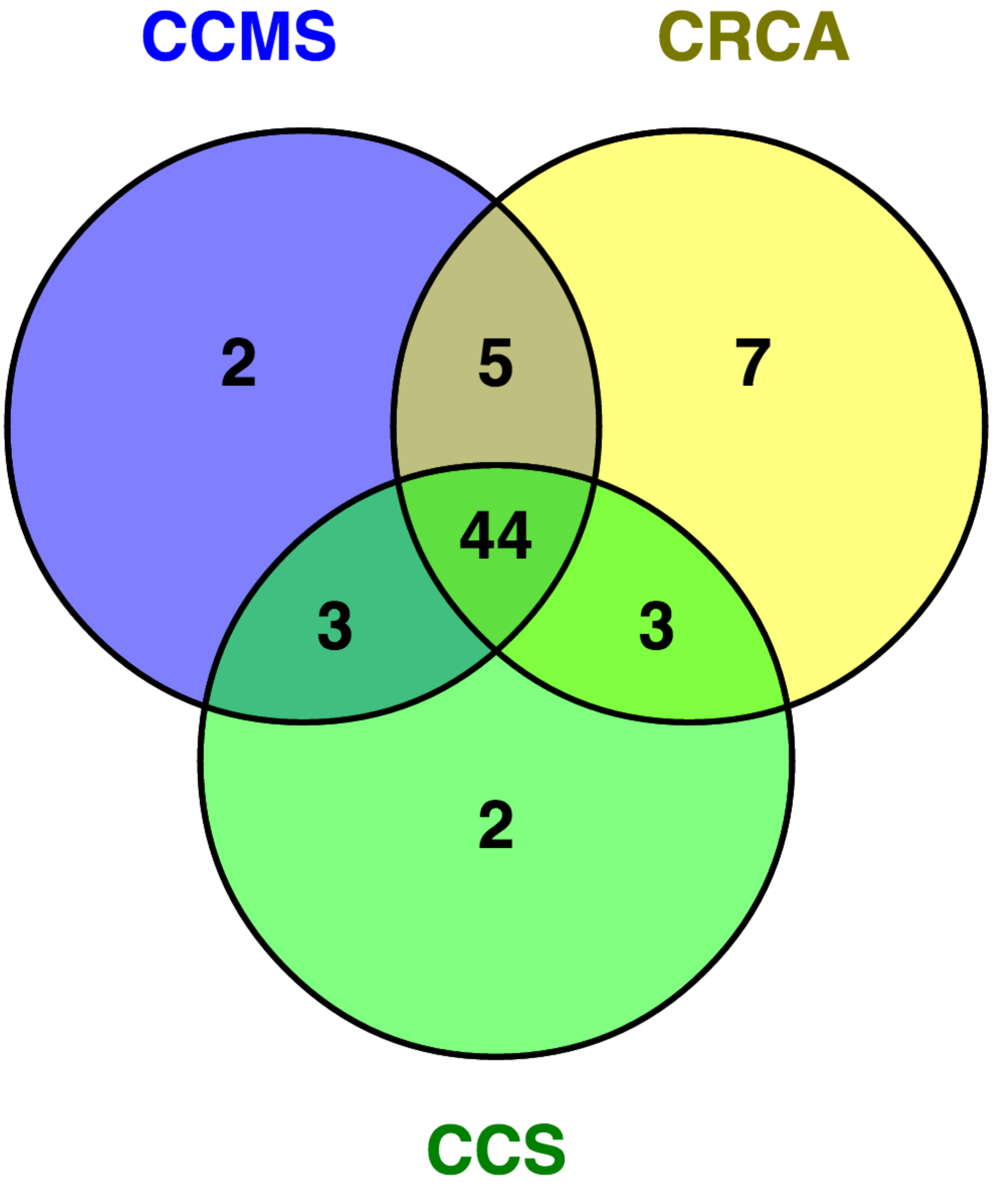

Supplementary Figure 1: Overlap between microRNAs with differential expression across subtypes defined by different classifiers. The Venn diagram shows the numbers of microRNAs differentially expressed in at least one subtype for each of the three classifiers, and the respective overlaps. Most microRNAs were detected as differentially expressed across subtypes in all three classification systems.

Supplementary Figure 2

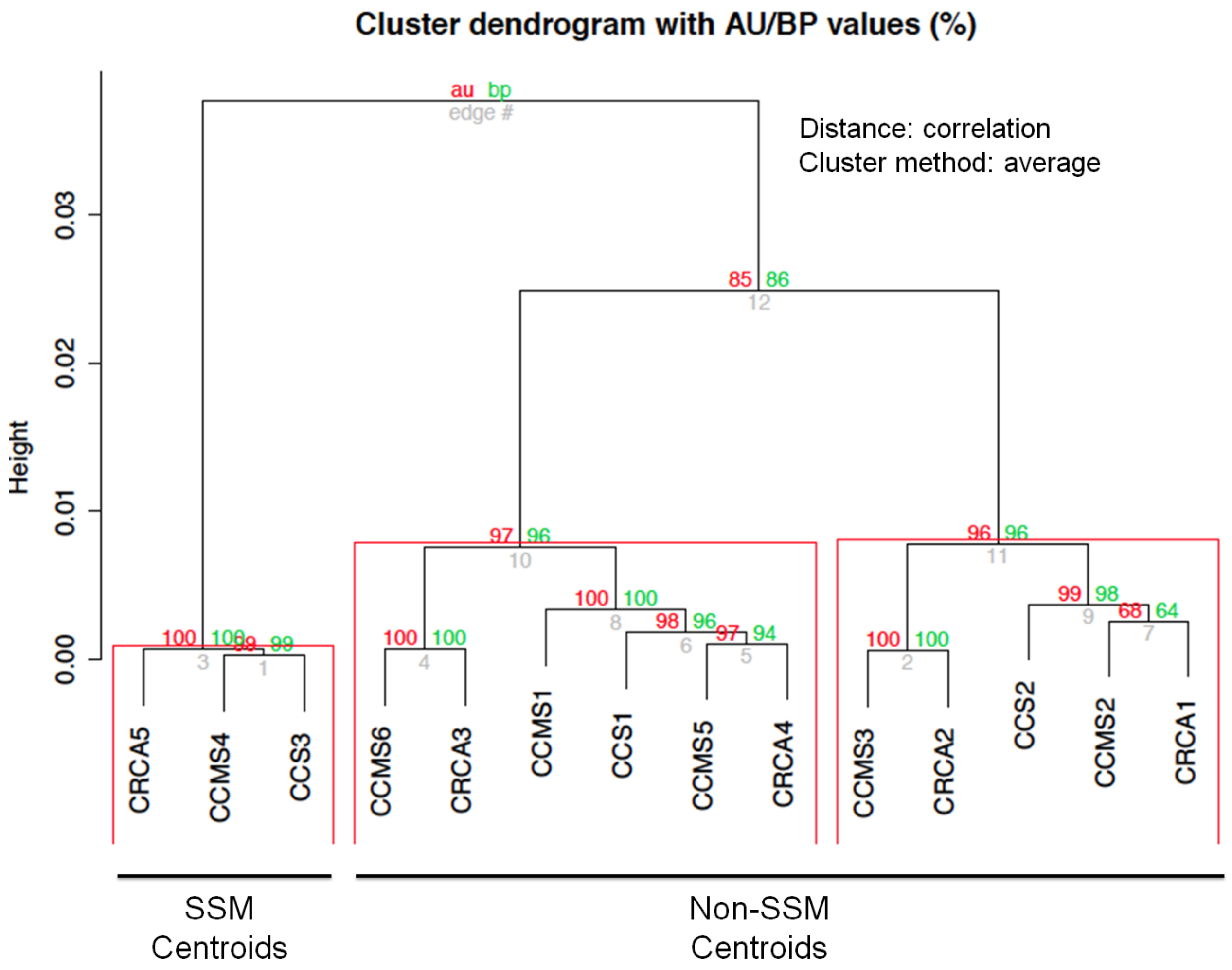

Supplementary Figure 2: Consensus clustering dendrogram as output of the pvclust CRAN package. Output of pvclust for a pvalue threshold of 0.05 applied in the microRNA expression dataset for 14 centroids (CRCA 1 to 5, CCS 1 to 3, CCMS 1 to 6) obtained considering the mean expression in each subtype of all the microRNAs differentially expressed in at least one subtype of one classifier. Red lines are drawn by the package to highlight groupings with more than 95% confidence. The dendrogram shows a first subdivision between SSM (left) and non-SSM centroids. The non-SSM centroids are then further partitioned in two subgroups: TA/Enterocyte (center) and Inflammatory/Goblet (left). The original hierarchical tree shown here has been redrawn in Figure 2 for graphic purposes.

Supplementary Table 1: Output of the MMRA pipeline - step II
List of microRNAs with a significant number of targets in the signature(s) for the subtype(s) in which they are differentially expressed. For each microRNAs is reported the classifier and the class in which the microRNA was differential and the sign. Then number of target mRNAs in the signature, hypergeometric p-value, Bonferroni adjusted p-value, observed on expected ratio and the minimum number of databases supporting microRNA target prediction used for the analysis

Mirna	Classifier	Subtype with miRNA differential expression	Up/Down Regulated	Gene Signature	Targets in signature	P-value	Bonferroni adjusted p-value	Observed / Expected	Min number of DBs predicting the interactions
hsa-miR-501-3p	CCMS	1	up	1 DOWN	9	5.96E-03	1.19E-02	2.74	2
hsa-miR-501-5p	CCMS	1	up	1 DOWN	9	1.16E-03	2.33E-03	3.51	2
hsa-miR-155	CCMS	2	up	2 DOWN	10	1.79E-04	3.40E-03	4.09	3
hsa-miR-223	CCMS	2	up	2 DOWN	5	2.19E-04	4.15E-03	9.19	4
hsa-miR-223	CCMS	2	up	2 DOWN	28	1.09E-07	2.06E-06	3.04	2
hsa-miR-181d	CCMS	2	down	2 DOWN	39	1.38E-05	5.24E-04	1.99	2
hsa-miR-375	CCMS	3	up	3 DOWN	9	4.02E-04	2.01E-03	4.07	3
hsa-miR-375	CCMS	3	up	3 DOWN	25	1.96E-04	9.81E-04	2.21	2
hsa-miR-31	CCMS	3	up	3 DOWN	37	5.95E-04	5.95E-03	1.76	2
hsa-let-7c	CCMS	4	up	4 UP	28	2.88E-05	1.15E-03	2.35	3
hsa-miR-1	CCMS	4	up	4 UP	24	2.16E-05	1.72E-03	2.60	3
hsa-miR-1	CCMS	4	up	4 UP	62	5.94E-08	4.75E-06	1.99	2
hsa-miR-130b	CCMS	4	down	4 UP	27	3.91E-05	1.56E-03	2.35	3
hsa-miR-130b	CCMS	4	down	4 UP	70	4.44E-11	1.77E-09	2.24	2
hsa-miR-135b	CCMS	4	down	4 UP	59	6.55E-08	2.62E-06	2.03	2
hsa-miR-141	CCMS	4	down	4UP	63	2.41E-06	1.93E-04	1.78	2
hsa-miR-143	CCMS	4	up	4 UP	15	8.02E-05	6.41E-03	3.21	3
hsa-miR-143	CCMS	4	up	4 UP	53	9.02E-06	7.21E-04	1.81	2
hsa-miR-148a	CCMS	4	down	4 UP	56	1.30E-07	5.22E-06	2.04	2
hsa-miR-153	CCMS	4	down	4 UP	58	3.41E-09	1.36E-07	2.22	2
hsa-miR-194	CCMS	4	down	4 UP	55	1.78E-07	7.11E-06	2.03	2
hsa-miR-19b	CCMS	4	down	4 UP	20	3.47E-05	1.39E-03	2.54	4
hsa-miR-19b	CCMS	4	down	4 UP	40	3.71E-09	1.48E-07	2.68	3
hsa-miR-19b	CCMS	4	down	4 UP	89	2.22E-16	8.88E-15	2.33	2
hsa-miR-200a	CCMS	4	down	4 UP	63	1.29E-06	1.03E-04	1.81	2
hsa-miR-203	CCMS	4	down	4 UP	13	1.13E-04	4.53E-03	3.46	4
hsa-miR-203	CCMS	4	down	4 UP	107	5.06E-14	4.05E-12	1.69	2
hsa-miR-29b	CCMS	4	down	4 UP	23	2.26E-08	9.02E-07	3.64	4
hsa-miR-29b	CCMS	4	down	4 UP	33	4.49E-07	1.80E-05	2.63	3
hsa-miR-29b	CCMS	4	down	4 UP	61	5.57E-09	2.23E-07	2.14	2
hsa-miR-33a	CCMS	4	down	4 UP	68	1.00E-11	4.00E-10	2.35	2
hsa-miR-362-3p	CCMS	4	down	4 UP	7	6.43E-05	2.57E-03	6.98	4
hsa-miR-362-3p	CCMS	4	down	4 UP	46	5.66E-06	4.53E-04	1.95	2
hsa-miR-375	CCMS	4	down	4 UP	11	4.01E-05	1.60E-03	4.46	3
hsa-miR-375	CCMS	4	down	4 UP	28	7.62E-05	3.05E-03	2.22	2
hsa-miR-429	CCMS	4	down	4 UP	30	1.31E-07	5.24E-06	2.96	3
hsa-miR-429	CCMS	4	down	4 UP	79	1.39E-09	1.11E-07	1.90	2
hsa-miR-155	CRCA	1	up	1 DOWN	49	5.65E-09	5.65E-08	2.41	2
hsa-miR-181d	CRCA	1	down	1 UP	60	3.91E-06	7.82E-05	1.58	2
hsa-miR-223	CRCA	1	up	1 DOWN	35	1.04E-05	1.04E-04	2.19	2
hsa-miR-31	CRCA	2	up	2 DOWN	8	1.95E-03	7.79E-03	3.57	3
hsa-miR-375	CRCA	2	up	2 DOWN	22	6.20E-05	2.48E-04	2.54	2
hsa-miR-1	CRCA	5	up	5 UP	52	1.73E-07	8.31E-06	2.07	2
hsa-miR-103	CRCA	5	down	5 UP	45	6.76E-06	6.49E-04	1.98	2
hsa-miR-130b	CRCA	5	down	5 UP	52	2.06E-07	9.90E-06	2.06	2
hsa-miR-135b	CRCA	5	down	5 UP	56	2.91E-10	1.40E-08	2.39	2
hsa-miR-141	CRCA	5	down	5 UP	52	8.29E-06	7.96E-04	1.82	2
hsa-miR-143	CRCA	5	up	5 UP	50	1.33E-07	6.36E-06	2.13	2
hsa-miR-148a	CRCA	5	down	5 UP	21	7.96E-06	3.82E-04	3.01	3
hsa-miR-148a	CRCA	5	down	5 UP	44	5.24E-06	2.51E-04	2.03	2
hsa-miR-153	CRCA	5	down	5 UP	44	1.36E-06	6.54E-05	2.13	2
hsa-miR-17	CRCA	5	down	5 UP	70	7.89E-09	7.58E-07	1.90	2
hsa-miR-194	CRCA	5	down	5 UP	54	1.67E-10	8.04E-09	2.48	2
hsa-miR-196a	CRCA	5	down	5 UP	36	2.58E-07	1.24E-05	2.54	2
hsa-miR-196b	CRCA	5	down	5 UP	32	6.40E-06	3.07E-04	2.35	2
hsa-miR-19b	CRCA	5	down	5 UP	33	4.63E-08	2.22E-06	2.71	3
hsa-miR-19b	CRCA	5	down	5 UP	65	2.31E-10	1.11E-08	2.18	2
hsa-miR-200b	CRCA	5	down	5 UP	73	2.54E-12	1.22E-10	2.22	2
hsa-miR-203	CRCA	5	down	5 UP	95	1.33E-15	1.28E-13	1.99	2
hsa-miR-20a	CRCA	5	down	5 UP	72	3.02E-09	2.90E-07	1.92	2
hsa-miR-218	CRCA	5	up	5 UP	56	3.23E-09	1.55E-07	2.24	2
hsa-miR-29b	CRCA	5	down	5 UP	18	1.15E-06	5.51E-05	3.88	4
hsa-miR-29b	CRCA	5	down	5 UP	53	3.55E-09	1.71E-07	2.30	2
hsa-miR-32	CRCA	5	down	5 UP	48	1.37E-06	6.60E-05	2.04	2
hsa-miR-33a	CRCA	5	down	5 UP	69	0.00E+00	0.00E+00	2.88	2
hsa-miR-33b	CRCA	5	down	5 UP	63	1.99E-14	9.54E-13	2.74	2
hsa-miR-429	CRCA	5	down	5 UP	71	4.98E-11	2.39E-09	2.12	2
hsa-miR-141	CCS	3	down	3 UP	18	1.19E-06	5.11E-05	3.46	2
hsa-miR-200a	CCS	3	down	3 UP	18	9.03E-07	3.88E-05	3.52	2

Supplementary Table 2: network reconstruction output as part of the MMRA pipeline - step III
For each microRNA is reported the number of links that constitute its regulon, and the minimum, 25th percentile, 50th percentile, 75th percentile and maximum of the mutual information distribution inside the regulon.

microRNA	Links	Mutual Information				
		Min	25 perc	50 perc	75 perc	Max
hsa-let-7c	565	0.1157699	0.139275	0.1641182	0.2313096	0.4424319
hsa-mir-1-2	333	0.1090721	0.1328165	0.1446437	0.1810169	0.4437721
hsa-mir-103-2	1492	0.1221676	0.151543325	0.17270345	0.211937175	0.3994345
hsa-mir-130b	760	0.120614	0.154788675	0.1956535	0.267832025	0.5019815
hsa-mir-135b	501	0.1129697	0.1362527	0.1475914	0.1643685	0.3209232
hsa-mir-141	653	0.1148168	0.1492166	0.1874171	0.254268	0.4000684
hsa-mir-143	358	0.1024992	0.1175124	0.12585265	0.14189275	0.2417733
hsa-mir-148a	555	0.1108313	0.1336147	0.1474636	0.17045335	0.2863548
hsa-mir-153-2	541	0.1120168	0.1324564	0.1432844	0.1605702	0.2513245
hsa-mir-155	307	0.1097238	0.1264053	0.1334794	0.14309815	0.3017963
hsa-mir-17	512	0.1170717	0.13850555	0.1566885	0.1771758	0.2615016
hsa-mir-181d	370	0.11124	0.1278833	0.13666995	0.15051935	0.2483261
hsa-mir-194-2	623	0.1145511	0.13890815	0.1513563	0.17156145	0.2781552
hsa-mir-196a-2	494	0.1199425	0.1474277	0.170625	0.210399775	0.3312043
hsa-mir-196b	381	0.1074994	0.1289608	0.1358197	0.1429325	0.3549173
hsa-mir-19b-2	440	0.1218705	0.1472143	0.1723403	0.228549075	0.3441318
hsa-mir-200a	596	0.1137988	0.146252	0.1736321	0.226540725	0.3784462
hsa-mir-200b	527	0.1169276	0.1391921	0.1569796	0.18197145	0.2777915
hsa-mir-203	557	0.1154339	0.1386004	0.1537655	0.1834574	0.2985793
hsa-mir-20a	569	0.1176115	0.1404244	0.1513587	0.168958	0.2682363
hsa-mir-218-2	280	0.1059009	0.121178875	0.12706095	0.1339836	0.2134157
hsa-mir-223	319	0.08833316	0.10575693	0.11535801	0.12650469	0.26370814
hsa-mir-29b-2	435	0.117454	0.15406375	0.1749873	0.20219345	0.2957062
hsa-mir-32	649	0.1172279	0.1499942	0.180131	0.2342764	0.4019606
hsa-mir-33a	620	0.1168592	0.1445292	0.16535845	0.19618815	0.3161642
hsa-mir-33b	511	0.1114853	0.1330975	0.1459859	0.167113	0.2615701
hsa-mir-362	717	0.1182087	0.1480641	0.1745561	0.2161976	0.3725019
hsa-mir-375	365	0.1083126	0.125765	0.1334354	0.1454737	0.3188456
hsa-mir-429	634	0.1199234	0.1461577	0.17345145	0.213945125	0.3548707
hsa-mir-501	521	0.1161564	0.1382991	0.1520166	0.1755833	0.2942241
hsa-mir-31	438	0.1091873	0.129785525	0.13814345	0.148484875	0.5715142

Supplementary Table 3: Output of the MMRA pipeline - step III
Results of the Master regulator analysis on each microRNA regulon. For each microRNA is reported the classifier subtype in which it is differential and the sign, the subtype signature significant in MRA and the MRA p-value.

Mirna	Classifier	miRNA sign	signature	MRA P-value
hsa-miR-29b	CCMS	DOWN4	UP4	1.21E-97
hsa-miR-429	CCMS	DOWN4	UP4	5.89E-92
hsa-miR-33a	CCMS	DOWN4	UP4	1.05E-91
hsa-miR-200a	CCMS	DOWN4	UP4	5.79E-86
hsa-miR-362-3p	CCMS	DOWN4	UP4	1.4E-85
hsa-miR-135b	CCMS	DOWN4	UP4	4.59E-84
hsa-miR-153	CCMS	DOWN4	UP4	1.47E-82
hsa-miR-203	CCMS	DOWN4	UP4	2.64E-82
hsa-miR-130b	CCMS	DOWN4	UP4	9.26E-82
hsa-miR-141	CCMS	DOWN4	UP4	2.95E-80
hsa-miR-148a	CCMS	DOWN4	UP4	1.72E-79
hsa-miR-194	CCMS	DOWN4	UP4	5.68E-77
hsa-miR-19b	CCMS	DOWN4	UP4	3.02E-65
hsa-miR-375	CCMS	DOWN4	UP4	1.77E-25
hsa-miR-501-5p	CCMS	UP1	DOWN1	1.31E-61
hsa-miR-501-3p	CCMS	UP1	DOWN1	1.31E-61
hsa-miR-155	CCMS	UP2	DOWN2	2.99E-06
hsa-miR-375	CCMS	UP3	DOWN3	6.71E-21
hsa-let-7c	CCMS	UP4	UP4	6.29E-80
hsa-miR-143	CCMS	UP4	UP4	1.75E-77
hsa-miR-1-2	CCMS	UP4	UP4	1.13E-35
hsa-miR-181d	CRCA	DOWN1	UP1	2.12E-26
hsa-miR-200b	CRCA	DOWN5	UP 5	4.92E-89
hsa-miR-20a	CRCA	DOWN5	UP 5	2.13E-85
hsa-miR-32	CRCA	DOWN5	UP 5	7.62E-84
hsa-miR-429	CRCA	DOWN5	UP 5	7.62E-84
hsa-miR-33a	CRCA	DOWN5	UP 5	5.57E-77
hsa-miR-194	CRCA	DOWN5	UP 5	6.54E-75
hsa-miR-17	CRCA	DOWN5	UP 5	5.48E-74
hsa-miR-130b	CRCA	DOWN5	UP 5	2.82E-73
hsa-miR-196a	CRCA	DOWN5	UP 5	3.68E-72
hsa-miR-29b	CRCA	DOWN5	UP 5	1.25E-67
hsa-miR-135b	CRCA	DOWN5	UP 5	9.47E-64
hsa-miR-153-2	CRCA	DOWN5	UP 5	4.79E-62
hsa-miR-148a	CRCA	DOWN5	UP 5	1.23E-60
hsa-miR-33b	CRCA	DOWN5	UP 5	3.37E-51
hsa-miR-19b	CRCA	DOWN5	UP 5	1.68E-45
hsa-miR-196b	CRCA	DOWN5	UP 5	3.25E-20
hsa-miR-103	CRCA	DOWN5	UP5	1.62E-92
hsa-miR-141	CRCA	DOWN5	UP5	9.47E-70
hsa-miR-203	CRCA	DOWN5	UP5	4.16E-58
hsa-miR-155	CRCA	UP1	DOWN1	3.11E-11
hsa-miR-223	CRCA	UP1	DOWN1	1.54E-09
hsa-miR-375	CRCA	UP2	DOWN2	1.24E-12
hsa-miR-143	CRCA	UP5	UP 5	1.65E-57
hsa-miR-218	CRCA	UP5	UP 5	6.42E-40
hsa-miR-1-2	CRCA	UP5	UP 5	3.17E-15
hsa-miR-200a	CCS	DOWN3	UP3	8.57E-23
hsa-miR-141	CCS	DOWN3	UP3	1.50E-21

Supplementary Table 4: Pathways and functions regulated by downregulation in CRC cell lines of miR-194, miR-200b, miR-203 and miR-429.
For each silenced microRNA, the table reports the results of GSEA analysis on significant gene sets from the Molecular Signature Database, plus the SSM-UP gene signature. The following columns report the gene set size, the enrichment score (ES), normalized enrichment score (NES), nominal p-value (NOM p-val), the FDR q-value and the family wise error rate (FWER) p-value.

MiRNA	msigDB signature	signature size	ES	NES	NOM p-val	FDR q-val	FWER p-val
miR-194	SSM-UP	55	0.5636264	2.0869489	0	0	0
miR-194	HALLMARK_TNFA_SIGNALING_VIA_NFKB	114	0.55700564	2.3553233	0	0	0
miR-194	HALLMARK_MYC_TARGETS_V1	191	0.50229067	2.2411664	0	0	0
miR-194	HALLMARK_MYC_TARGETS_V2	53	0.5594072	2.0401158	0	0.005675952	0.045
miR-200b	SSM-UP	55	0.5688495	2.1029637	0	0	0
miR-200b	HALLMARK_TNFA_SIGNALING_VIA_NFKB	114	0.5439759	2.305035	0	0	0
miR-200b	NABA_ECM_REGULATORS	45	0.5946715	2.1171129	0	0.002587928	0.011
miR-200b	HALLMARK_EPITHELIAL_MESENCHYMAL_TRANSITION	59	0.5638333	2.110836	0	0.002737465	0.014
miR-203	SSM-UP	55	0.41407347	1.5512599	0.016491754	0.016491754	0.011
miR-203	REACTOME_TGF_BETA_RECEPTOR_SIGNALING_ACTIVATES_SMADS	16	0.68752563	1.9410322	0.001766785	0.05258462	0.209
miR-203	REACTOME_DOWNREGULATION_OF_TGF_BETA_RECEPTOR_SIGNALING	15	0.6965812	1.9252508	0.001658375	0.048333574	0.264
miR-203	HALLMARK_E2F_TARGETS	174	-0.3851316	-1.9533771	0	0.062436868	0.284
miR-203	HALLMARK_G2M_CHECKPOINT	159	-0.39014986	-1.9490144	0	0.053237937	0.298
miR-429	SSM-UP	55	0.5333889	1.9539242	0	0	0
miR-429	HALLMARK_TNFA_SIGNALING_VIA_NFKB	114	0.57472944	2.3985708	0	0	0
miR-429	HALLMARK_INTERFERON_GAMMA_RESPONSE	101	0.53009456	2.136673	0	0.001208048	0.007
miR-429	HALLMARK_INFLAMMATORY_RESPONSE	70	0.5424787	2.0767472	0	0.002633725	0.026

Supplementary Table 5: Output of FDR computation for the MMRA pipeline - step I.
In the first two columns the list of all possible couples (p-value threshold, fold change threshold) are reported. For each couple are reported the classifier system, the number of significant microRNAs and the obtained FDR. The rows with the best FDR for each classifier are highlighted in orange.

p.value threshold	Fold Change threshold	Classifier	number of significant microRNAs	Average DE miRNAs in 1000 random sample permutations	Global FDR
0.05	1.5x	CCS	134	0.3082	0.2%
0.05	2x	CCS	53	0.3604	0.7%
0.05	2.5x	CCS	25	0.055	0.2%
0.01	1.5x	CCS	133	1.729	1.3%
0.01	2x	CCS	53	0.265	0.5%
0.01	2.5x	CCS	25	0.04	0.2%
0.001	1.5x	CCS	132	0.99	0.8%
**0.001**	**2x**	**CCS**	**52**	**0.052**	**0.1%**
0.001	2.5x	CCS	25	0.025	0.1%
0.05	1.5x	CCMS	156	10.452	6.7%
0.05	2x	CCMS	57	1.824	3.2%
0.05	2.5x	CCMS	27	0.864	3.2%
0.01	1.5x	CCMS	152	5.472	3.6%
0.01	2x	CCMS	57	0.684	1.2%
0.01	2.5x	CCMS	25	0.41	1.6%
0.001	1.5x	CCMS	143	1.1726	0.8%
**0.001**	**2x**	**CCMS**	**54**	**0.351**	**0.7%**
0.001	2.5x	CCMS	24	0.1512	0.6%
0.05	1.5x	CRCA	159	6.678	4.2%
0.05	2x	CRCA	60	1.14	1.9%
0.05	2.5x	CRCA	33	0.297	0.9%
0.01	1.5x	CRCA	158	4.266	2.7%
0.01	2x	CRCA	60	0.462	0.8%
0.01	2.5x	CRCA	33	0.231	0.7%
0.001	1.5x	CRCA	151	1.51	1.0%
**0.001**	**2x**	**CRCA**	**59**	**0.118**	**0.2%**
0.001	2.5x	CRCA	33	0.0561	0.2%

Supplementary Table 6: Output of FDR computation for the MMRA pipeline - step II.
In the first two columns the list of all possible couples (Hypergeometric p-value threshold,observed/expected threshold) are reported. For each couple are reported the classifier system, the number of significant microRNAs and the obtained FDR. The rows with the best FDR for each classifier are highlighted in orange.

Hypergeometric p-value threshold	Observed/Expected Threshold	Classifier	number of significant microRNAs	FDR
0.05	1.5	CRCA	31	17.6%
0.05	2	CRCA	25	17.4%
0.05	2.5	CRCA	14	22.0%
0.01	1.5	CRCA	29	15.0%
0.01	2	CRCA	24	15.4%
0.01	2.5	CRCA	12	18.9%
**0.001**	**1.5**	**CRCA**	**26**	**12.8%**
0.001	2	CRCA	20	16.0%
0.001	2.5	CRCA	7	29.3%
0.05	1.5	CCMS	30	17.0%
0.05	2	CCMS	21	19.8%
0.05	2.5	CCMS	15	21.1%
**0.01**	**1.5**	**CCMS**	**23**	**16.8%**
0.01	2	CCMS	18	19.4%
0.01	2.5	CCMS	11	26.7%
0.001	1.5	CCMS	17	19.0%
0.001	2	CCMS	10	28.9%
0.001	2.5	CCMS	4	64.8%
0.05	1.5	CCS	4	47.4%
0.05	2	CCS	4	44.7%
0.05	2.5	CCS	4	47.1%
0.01	1.5	CCS	2	46.8%
0.01	2	CCS	2	46.8%
0.01	2.5	CCS	2	38.4%
0.001	1.5	CCS	2	14.9%
0.001	2	CCS	2	18.3%
**0.001**	**2.5**	**CCS**	**2**	**9.1%**

Supplementary Note

MMRA validation and comparisons with alternative procedures

To test the robustness and reliability of the MMRA pipeline we performed the following analyses:

1. Pipeline validation in two independent datasets
2. Comparisons with variants of the pipeline
3. Comparison with other pipelines and methods

For the sake of space, all the comparisons presented here were made for the CCMS classifier[1] because it is the one with the highest number of signature genes per subtype and therefore the one giving the largest lists of candidate microRNAs in output. We want to emphasize that the absence or low size of a gene signature is not an obstacle for the pipeline application. In fact, there are many procedures that can be applied for signature construction starting from an annotated expression dataset. However, it is better to reconstruct the gene signature on a dataset independent from the one that is used to apply the MMRA pipeline, to avoid overfitting and to guarantee that the defined signature is effectively well representative of the studied phenotypes.

1. Pipeline validation in two independent datasets

We considered testing the MMRA pipeline on an independent dataset, but we weren't able to find a paired mRNA/microRNA CRC expression dataset of at least 100 samples needed to apply the pipeline. Therefore we randomly divided the TCGA dataset in two subsets using the R function "sample()"[2,3]. We compared the outputs obtained in the two datasets at each step of the pipeline. After the first step, we obtained 55 differentially expressed microRNAs in dataset one and 67 in dataset two, with an intersection of 44 microRNAs (best / worst validation rate = 80% / 66%). After the second step of target transcript enrichment analysis we obtained 22 microRNAs in dataset one and 18 in dataset two, with an intersection of 15 microRNAs (best / worst validation rate = 83% / 69%). Finally, after the network analysis phase, we obtained 17 microRNAs in dataset one and 14 microRNAs in dataset two, with an intersection of 11 microRNAs (best / worst validation rate = 79% / 65%). These results allowed us to estimate that in suboptimal conditions due to reduced size of the data subsets, the independent validation rate was between 65% and 80% at all

steps of the analysis. Notably, all four microRNAs experimentally validated on cell lines were included in the 11 cross-validated microRNAs, showing that biologically relevant interactions emerge repeatedly also when lower size datasets are employed for MMRA.

2. Comparison with variants of the pipeline

Five variations of the MMRA pipeline were implemented and compared with the original procedure that, when applied on CCMS-classified samples, yielded 13 microRNA/subtype associations, of which 5 (40%) were validated in cell lines. Of note, the comparison was made considering microRNA/subtype associations, not only on the number of microRNAs: one microRNA may have more than one subtype association.

- Alternative pipeline 1: microRNA differential expression analysis followed only by target enrichment analyses, i.e. only steps 1 and 2 of MMRA. Given that steps 3 and 4 of MMRA filtered out only 7 microRNAs, we considered their removal from the pipeline. This yielded 24 microRNA/subtype associations, of which only 6 (25%) validated in cell lines. All 13 associations identified by MMRA were of course also found here. This pipeline variant can therefore be considered slightly more sensitive and noticeably less specific.

- Alternative pipeline 2: microRNA differential expression analysis and target enrichment analyses followed by Stepwise linear regression, to test the contribution of the network analysis step to the performances of MMRA. This variant yielded 12 microRNA/subtype associations, of which only 3 (25%) validated in cell lines. Of the 13 associations identified by MMRA, 8 were also found by this pipeline, of which 1 (13%) validated in cell lines. This pipeline can therefore be considered less sensitive and substantially less specific.

- Alternative pipeline 3: microRNA differential expression analysis followed by GSEA analysis on genes ranked by their correlation with the microRNA. The GSEA step is aimed at verifying, without the use of thresholds, whether, among all expressed genes, microRNA predicted targets belonging to the associated subtype gene signature are significantly more anticorrelated or correlated with the microRNA across the whole dataset. This variant yielded 69 microRNA/subtype associations, of which only 19 (28%) validated in cell lines. All 13 associations identified by MMRA were also found by this pipeline, that can therefore be considered more sensitive but considerably less specific.

- Alternative pipeline 4: microRNA differential expression analysis and target enrichment analyses followed by GSEA analysis to verify if the genes belonging to the signature associated to the microRNA are significantly more anticorrelated or correlated with the microRNA in respect to all the expressed genes. This variant yielded 23 microRNA/subtype associations, of which only 3 (13%) validated in cell lines. Of the 13

associations identified by MMRA, 11 were also found by this pipeline, of which 3 (27%) validated in cell lines. This pipeline can therefore be considered slightly more sensitive and substantially less specific.

- Alternative pipeline 5: microRNA differential expression analysis followed only by stepwise linear regression analysis. This yielded 10 microRNA/subtype associations, of which only 2 (20%) validated in cell lines. 2 of the 13 associations identified by MMRA were also found here. This pipeline variant can therefore be considered slightly less sensitive and noticeably less specific.
- Alternative pipeline 6: microRNA differential expression analysis followed by stepwise linear regression analysis restricted to the signature genes that have a miRNA-target relationship. This variant yielded 7 microRNA/subtype associations, of which only 2 (29%) validated in cell lines. 3 of the 13 associations identified by MMRA were also found by this pipeline, that can therefore be considered considerably less sensitive and less specific.

These results show that every tested change to the MMRA pipeline always reduced its specificity, in some cases increasing sensitivity and in others also reducing sensitivity.

3. Comparisons with other pipelines and methods

- Alternative pipelines. We considered for comparison simpler pipelines originally developed to discover microRNA-mRNA interactions dysregulated in cancer vs. normal tissue. It must be noted that the differences between normal and transformed tissues are much wider than those between tumor subtypes. Two such pipelines are available online: The first, by Fu and colleagues[4], is also at the basis of other pipelines, and involves microRNA and mRNA differential expression analysis, followed by anticorrelation analysis, leading to the selection of anticorrelated microRNA/mRNA pairs in which the mRNA is also a predicted target of the microRNA; the second, by Pizzini and colleagues[5], follows the basic steps of the Fu pipeline, but in the final output also the microRNA-mRNA pairs in which the mRNA is not significantly differentially expressed are reported. Moreover, this pipeline also integrates the effects of transcription factors on these interactions, which adds an additional variable to the interaction analysis. For this reason we selected for comparison the basic Fu pipeline. In our dataset, the output of this pipeline was composed of broad lists of microRNA-mRNA interactions. Each microRNA was frequently associated to more than one subtype. No prioritization was made in the output based on the potentiality of the microRNA to be driver of the associated class. Finally, this kind of pipeline takes in account only those microRNA-target interactions supported by

anticorrelation, even if has been recently observed that microRNAs can act also indirectly through the regulation of silencing complexes[6]. Overall, the number of microRNAs identified by the two pipelines is comparable, but the number of microRNA/class associations was higher for the Fu pipeline, because it associated each microRNA to more than one subtype in the same classifier. This in principle could happen also for MMRA, but it did not occur in the present analysis. Therefore, while the MMRA pipeline identified 13 microRNAs, each with one subtype association, the Fu pipeline identified 40 microRNA/subtype associations involving 23 microRNAs. The fraction of associations validated in cell lines was reduced to 32% in the Fu pipeline. However, of the 13 interactions identified by MMRA, 8 were also identified by the FU pipeline, and the validation rate in cell lines for these associations raised to 63% (5 out of 8). This result indicates that combined use of the two pipelines could result in shorter but more reliable lists of microRNA/subtype associations.

- Alternative method. The last question that we addressed is if a simple miRNA differential expression analysis followed by analysis of anticorrelation with subtype signature genes could bring to the identification of the same microRNAs obtained by MMRA. As a first point, the output of such alternative procedure would be a list of microRNAs ranked by their anticorrelation to subtype signature genes, after which the problem of choosing significance thresholds and estimating FDR would have to be addressed. To avoid choosing thresholds, we verified whether the top subtype signature anti-correlated MiRs were different from those identified by MMRA. As described above, MMRA applied to CCMS classification and signatures identified 13 microRNAs of which 12 associated to the CCMS4-UP signature and one associated to the CCMS1-DOWN signature, where “UP” and “DOWN” mean up-regulation and down-regulation in the subtype, respectively. Given that the microRNA associated to the CCMS1-DOWN signature is also the only one differentially expressed in this class, correlation analysis would not allow comparing ranks. For this reason the comparison was performed only for CCMS4-UP-associated microRNAs. The number of differentially expressed microRNAs in CCMS4 subtype is 38. For each differentially expressed microRNA, we computed the mean correlation with the genes of the CCMS4-UP signature. Then, microRNAs were ranked by increasing average correlation values, and the 12 microRNAs most anticorrelated with the CCMS4-UP signature were compared with the 12 microRNAs associated by MMRA to the CCMS4-UP signature. Only 7 microRNAs were present in both lists. Moreover miR-203, that we functionally validated in cell lines, was in position 17 of the anticorrelation ranking,

therefore it would not be selected according to this kind of procedure. Interestingly the validation rate in cell lines of this alternative method is 33% while the validation rate of MMRA output restricted only to CCMS4 is 42%. This shows that a simple analysis of differential expression followed by correlation analysis couldn't capture the relationship between master MiRs and subtype profiles identified by MMRA. In fact some of the most anticorrelated microRNAs are not significant in the MMRA pipeline, and vice versa.

Supplementary References

1. Marisa, L. *et al.* Gene expression classification of colon cancer into molecular subtypes: characterization, validation, and prognostic value. *PLoS Med* **10**, e1001453 (2013).
2. Becker, R.A., Chambers, J. M. and Wilks, A. R. *The New S Language*, (1988).
3. Ripley, B.D. *Stochastic Simulation*, (1987).
4. Fu, J. *et al.* Identifying microRNA-mRNA regulatory network in colorectal cancer by a combination of expression profile and bioinformatics analysis. *BMC Syst Biol* **6**, 68 (2012).
5. Pizzini, S. *et al.* Impact of microRNAs on regulatory networks and pathways in human colorectal carcinogenesis and development of metastasis. *BMC Genomics* **14**, 589 (2013).
6. Fabbri, M., Calore, F., Paone, A., Galli, R. & Calin, G.A. Epigenetic regulation of miRNAs in cancer. *Adv Exp Med Biol* **754**, 137-48 (2013).
